# Bioactive Materials for Bone Regeneration: Biomolecules and Delivery Systems

**DOI:** 10.1021/acsbiomaterials.3c00609

**Published:** 2023-08-16

**Authors:** Aleksandra Szwed-Georgiou, Przemysław Płociński, Barbara Kupikowska-Stobba, Mateusz M. Urbaniak, Paulina Rusek-Wala, Konrad Szustakiewicz, Paweł Piszko, Agnieszka Krupa, Monika Biernat, Małgorzata Gazińska, Mirosław Kasprzak, Katarzyna Nawrotek, Nuno Pereira Mira, Karolina Rudnicka

**Affiliations:** † Department of Immunology and Infectious Biology, Faculty of Biology and Environmental Protection, University of Lodz, Lodz 90-136, Poland; ‡ Biomaterials Research Group, Lukasiewicz Research Network - Institute of Ceramics and Building Materials, Krakow 31-983, Poland; § The Bio-Med-Chem Doctoral School, University of Lodz and Lodz Institutes of the Polish Academy of Sciences, University of Lodz, Lodz 90-237, Poland; ∥ Department of Polymer Engineering and Technology, Faculty of Chemistry, Wroclaw University of Technology, Wroclaw 50-370, Poland; ⊥ Faculty of Process and Environmental Engineering, Lodz University of Technology, Lodz 90-924, Poland; # iBB-Institute for Bioengineering and Biosciences, Department of Bioengineering, Instituto Superior Técnico, Universidade de Lisboa, Lisboa 1049-001, Portugal; ▲ Associate Laboratory i4HB-Institute for Health and Bioeconomy at Instituto Superior Técnico, Universidade de Lisboa, Lisboa 1049-001, Portugal; ■ Instituto Superior Técnico, Universidade de Lisboa, Lisboa 1049-001, Portugal

**Keywords:** bioactive materials, biomolecules, biomolecule delivery systems, bone healing, bone regeneration, biomaterials, composites, scaffolds

## Abstract

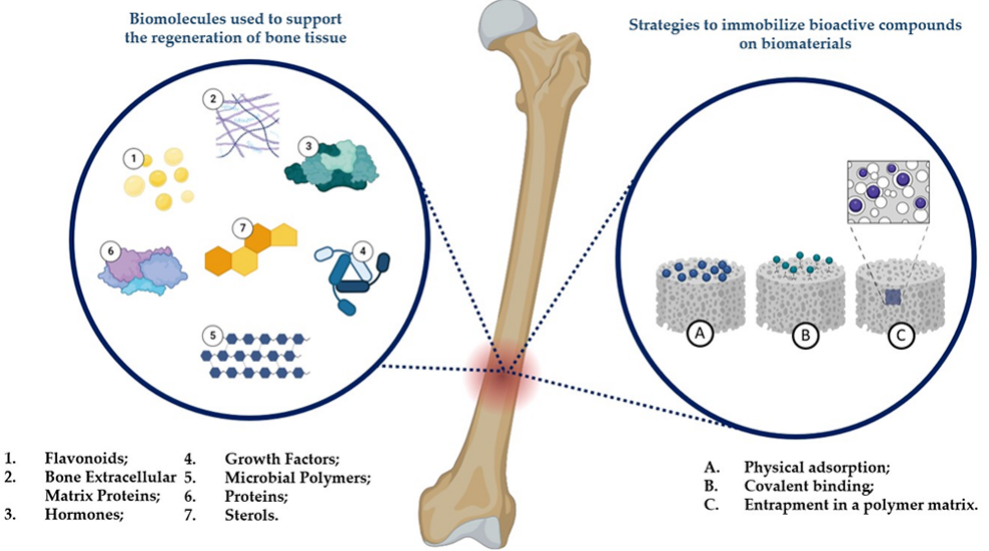

Novel tissue regeneration strategies are constantly being developed worldwide. Research on bone regeneration is noteworthy, as many promising new approaches have been documented with novel strategies currently under investigation. Innovative biomaterials that allow the coordinated and well-controlled repair of bone fractures and bone loss are being designed to reduce the need for autologous or allogeneic bone grafts eventually. The current engineering technologies permit the construction of synthetic, complex, biomimetic biomaterials with properties nearly as good as those of natural bone with good biocompatibility. To ensure that all these requirements meet, bioactive molecules are coupled to structural scaffolding constituents to form a final product with the desired physical, chemical, and biological properties. Bioactive molecules that have been used to promote bone regeneration include protein growth factors, peptides, amino acids, hormones, lipids, and flavonoids. Various strategies have been adapted to investigate the coupling of bioactive molecules with scaffolding materials to sustain activity and allow controlled release. The current manuscript is a thorough survey of the strategies that have been exploited for the delivery of biomolecules for bone regeneration purposes, from choosing the bioactive molecule to selecting the optimal strategy to synthesize the scaffold and assessing the advantages and disadvantages of various delivery strategies.

## Introduction

Advanced strategies for the regeneration of various tissue defects continue to emerge in plastic and reconstructive medicine and in dentistry. Millions of individuals suffer from bone loss each year; and although bone tissue naturally possesses high regeneration potential, its capacity to repair itself can be limited by secondary factors such as the extent of bone loss, the age and sex of the individual, and comorbidities. Bone defects typically resulting from extensive trauma, tumors, infections, inflammation, or degenerative disorders can be healed with advanced treatments.

The need for hard tissue regeneration biomaterials has substantially increased as the world’s population ages. Bone fractures, defects, and nonunions are a global healthcare problem. Moreover, fragility fractures, typically occurring in osteoporosis, located in wrists, hips, and vertebrae, can often be debilitating, put patients at an increased risk for a subsequent fracture, and can even be fatal among older adults.^1^ Worldwide, women over 50 years old have a 9.8–22.8% risk of fragility fractures.^2^ The Bone Health and Osteoporosis Foundation estimates that 3 million fractures and $25.3 billion in direct healthcare costs will arise annually by 2025. The total cost of care associated with osteoporotic fractures and nonunion fractions will reach $95 billion in 2040.^3^

The gold standard—allografts—is impeded by potential infection, limited availability, and a high nonunion rate with host tissues. Biomaterials that mimic bone tissue are becoming critical components of reconstructive approaches for cases where autogenous bone grafts are not obtainable. There are currently a large variety of bone matrices that can be used to treat bone loss. Among them, there are materials delivering natural or synthetic materials that are compatible with regenerative medicine. New matrices are being developed each year, and these are commonly coupled with growth factors (GFs) and other bone growth stimulants and infused with antibiotics to lower the risk of infection. On the other hand, the World Health Organization identifies antibiotic resistance as one of the biggest threats to global health, and their overuse in prophylaxis for bone should be limited.^4^ The search for composites with optimal biocompatibility and osteointegrative, osteoconductive, and osteoinductive properties is ongoing. Expectedly, such new generation of biomaterials must also allow the efficient recruitment of mesenchymal stem cells (MSCs) that will colonize the scaffold and differentiate into bone tissue with the desired shape, form, and durability.

**Figure 1:**
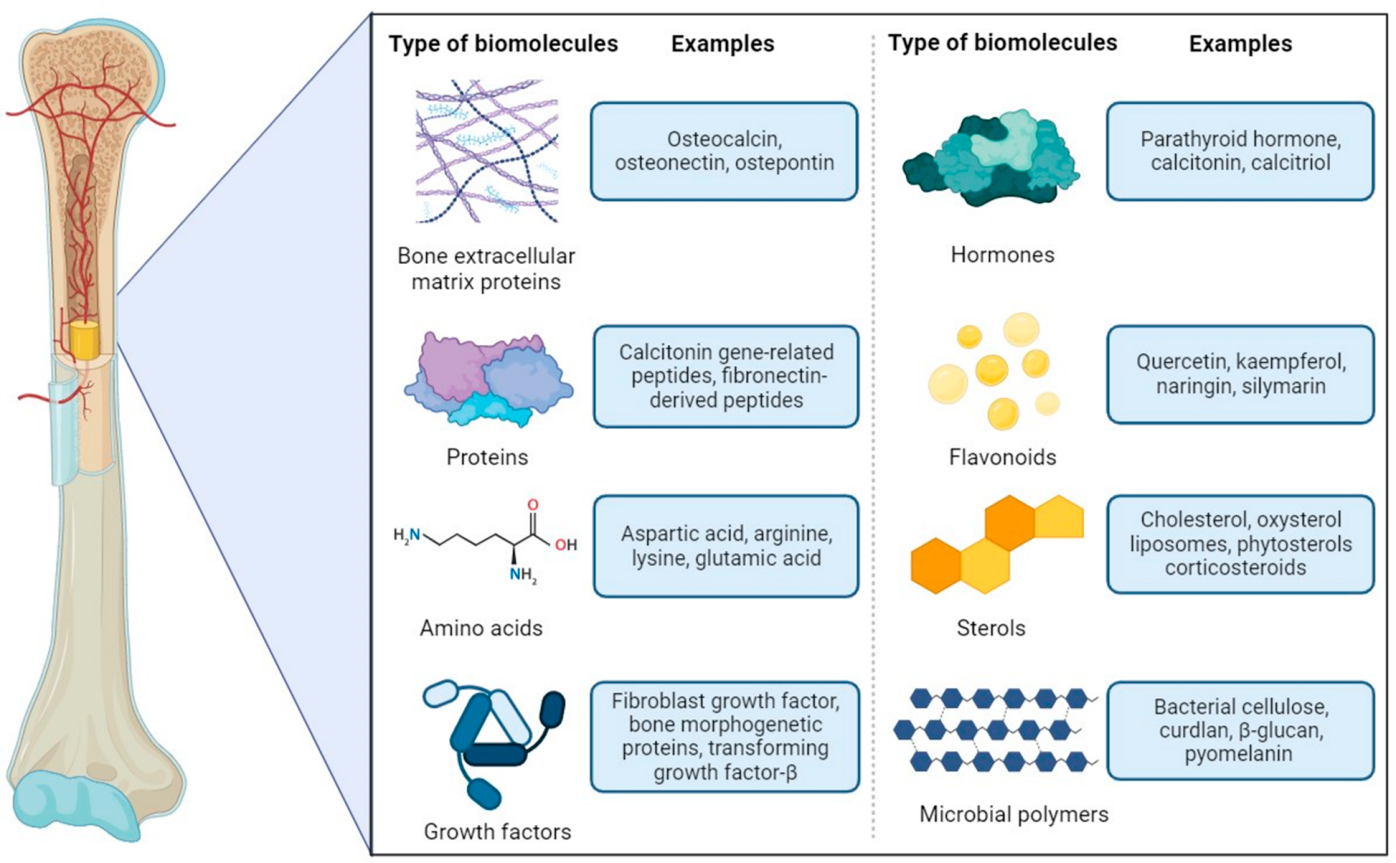
Types and examples of biomolecules used to support the regeneration of bone tissue.

This paper reviews recent developments in using biomaterials and constructs for hard tissue repair and regeneration (Figure 1). The multidisciplinary group of chemists, material engineers, molecular biologists, biotechnologists, and microbiologists worked together to explore recent advances *in vitro* and *in vivo* research on the efficiency of bioactive molecules, their delivery platforms, and methods to produce polymeric materials. The first section of this review concentrates on bioactive components that support biocompatibility and bone regeneration using bone extracellular matrix (ECM), hormones, plant-derived flavonoids and sterols, peptides, amino acids, and microbial polymers. In the second section, we explore selected methods and pathways to produce materials and scaffolds, including polymers, inorganic fillers, and solvent-free techniques. Finally, we present the delivery methods that ensure the activity of the biomolecules, e.g., obtained by surface functionalization, controlled and stimuli-driven delivery, and gene-delivery systems. The last sections discuss recent advances, highlighting challenges and possible solutions in the design and application of biomaterials in bone tissue engineering.

## Biomolecules Used for Bone Regeneration

### Bone Extracellular Matrix (ECM) Proteins

Bone tissue mainly comprises cells mounted in a biomineral matrix.^5^ The extracellular matrix (ECM) is a complex and constantly changing biological environment with specific mechanical and biochemical properties. The ECM plays a crucial role in regulating cell adhesion, proliferation, responses to growth factors, and differentiation, ultimately affecting the mature bone’s functional characteristics. Osteoblast-lineage cells, including MSCs, osteoblasts, and osteocytes, can produce new bone when stimulated by the bone ECM, whereas osteoclasts can absorb bone.^6^ The structure of bone’s biomineral scaffold consists of around 70% of inorganic calcium hydroxyapatite (HA) crystals—Ca_10_(PO_4_)_6_(OH)_2_. The remaining 30% comprises organic elements, with collagens being the predominant proteinaceous components followed by noncollagenous proteins (NCPs), lipids, proteoglycan molecules, and other bone matrix proteins.^7^ Bone ECM proteins are vital factors in bone tissue’s mechanical strength and adhesive characteristics of bone tissue.^8^ Moreover, ECM mineralization is an essential and critical step in bone repair and reconstruction. Matrix mineralization, and the synthesis and secretion of type I collagen and NCPs by osteoblasts, are hallmarks of bone formation. The scaffold frame formed by the deposition of collagen fibers constitutes the structural basis of bone mineralization, whereas NCPs are involved in HA deposition. The ECM also plays a critical role in regulating cell adhesion, movement, and migration.^9^

As said above, collagens are the main structural proteins present in the ECM of bone tissue, constituting over 20% of bone mass and up to 90% of bone’s organic matrix.^10^ Type I collagen is the main collagen type implicated in bone mineralization; however, small quantities of type V collagen bound to HA crystals typically found in bone tissue. Collagen type III alongside with type V are thought to influence fibrillogenesis and fiber diameter of type I collagen. Collagen matrix organization is critical to maintain the optimal mechanical properties of the bone, and abnormalities in collagens hierarchical structures are associated with serious conditions, including osteogenesis imperfecta and Paget’s disease.^11^ Collagens, especially type I, are often used in bone replacement composites to improve their structural and functional properties.

Osteocalcin (OC) is among the most abundant NCPs in the bone matrix. OC is a relatively small matrix protein dependent on vitamins D and K. Each OC molecule contains three γ-carboxyglutamic acid molecules with a strong affinity for Ca^2+^. The γ-carboxyglutamic acid moieties are responsible for the high affinity of OC for HA.^12,13^ Mature osteoblasts produce the 49 amino acids long OC protein, which is directly involved in the regulation of bone density. Moreover, OC promotes bone mineralization and formation and attracts osteoclast progenitors.^14,15^ OC is believed to influence the early stages of bone healing and regulate the activity of osteoblasts and hydroxyapatite binding.^16−18^ Previous studies demonstrated that OC could successfully enhance the adhesion of osteoblast-like cells on the surface of HA/collagen I containing materials *in vitro*.^19^ A study by Rammelt et al.^20^ noted the significantly faster replacement of woven bone by lamellar bone when HA/collagen I implants were enriched with OC compared to unmodified implants. This result indicates that OC accelerates *de novo* bone formation rather than increasing the formation of new bone.^20^

Another important NCP that can be used to tune the bone regeneration process is osteonectin, also known as secreted protein, acidic and rich in cysteine (SPARC) or BM-40.^21,22^ Osteonectin has a high affinity for collagen I and HA.^23^ Like OC, osteonectin is involved in bone matrix mineralization.^24^ It is also a modular protein that regulates cell behavior and can influence tissue remodeling, repair, development, and cell turnover.^25^ A study by Zhu et al.^26^ provided evidence that osteonectin regulates the mineralization process in osteoblasts and is a crucial component of the p38 signaling pathway. Osteonectin can also modulate bone density. Thus, it holds tremendous potential as a point for bone reconstruction and regeneration interventions.^26^

Osteopontin (OPN) is another ECM NCP that can be used in the bone regeneration process. OPN is a member of the small integrin-binding ligand family N-glycosylated proteins, along with bone sialoprotein (BSP), dentin matrix protein 1, and matrix extracellular phosphoprotein. OPN mediates the attachment of bone cells to the mineral crystal structure and regulates bone resorption and calcification. Moreover, OPN is active in biological processes, such as wound healing, immunological reactions, tumorigenesis, atherosclerosis, and angiogenesis.^27^ McKee, Pedraza, and Kaartinen^28^ suggested that OPN may have an essential role in bone regeneration processes after surgical cutting when bone debris (powder) is cleared by macrophage phagocytosis after OPN opsonization and a cement line (plane) is formed at the margins of the wound that integrates the newly repaired bone with the existing drilled bone.^28^ Furthermore, other studies have shown that the so-called “glue” effect of some NCPs (OPN, OC, osteonectin) plays a significant role in promoting the integration of collagen fibrils and apatites.^7,29^

Sun et al.^30^ indicated that NCPs could be extracted from bone ECM and successfully coupled to the surfaces of nanofibrous (NF) gelatin scaffolds. *In vitro* studies revealed that NF-gelatin-NCP scaffolds promoted the osteoblasts’ proliferation, differentiation, and mineralization. Importantly, *in vivo* calvarial bone defect experiments demonstrated that the scaffolds containing NCPs could recruit more host cells to the defect and regenerate more bone than the control scaffolds at 6 weeks postimplantation. Thus, integrating NCPs into scaffolds is a promising strategy for improving the bone regeneration process.^30^

Other important protein components of ECM include positive (e.g., Periostin) and negative (matrix Gla protein, bone Gla protein) regulators of bone formation, mineralization, and remodeling (thrombospondins and R-spondins);^6^ however, their potential application in functionalization of bone replacement scaffolds has yet to be fully investigated.

### Peptides

Bone tissue engineering (BTE) and research on peptides have expanded significantly in recent years. The outcomes of these extensive studies have shown that several peptides can support and stimulate the bone healing response.^31^ The practical advantage of using peptides over proteins is that they can be produced with precise control of their chemical structures. Moreover, compared to proteins, peptides are also more resistant to denaturation caused by temperature or pH variations than proteins and are easier to manipulate during grafting. Bioactive peptides that can promote the regeneration of local bone defects can be mainly divided into ECM-derived peptides, and bone morphogenetic protein (BMP)-derived peptides.^32^

The most-studied ECM-derived peptides contain signaling domains, as they can connect to receptors on the surface of the cell membrane.^33^ Selected examples of ECM-derived peptides that have been used in bone repair and regeneration studies are shown in Table 1.

**Table 1:** *In Vitro* and *In Vivo* Studies That Have Reported the Osteogenic Effects of Bioactive Peptides in Bone Regeneration^34^

**Bioactive peptide** [Reference(s)]	**Composition** [number of amino acids]	**Binding site/potential pathway(s)**	**Genes or proteins up-/downregulated by peptide**	**Function**
**ECM-Derived Peptides**				
PepGen P-15 (P-15) [^385−388^]	15	Type I collagen binding sites	Upregulated: ALP, BMP-2, BMP-7; Runx2, COL1, OSTRX BSP, and integrin a2	Promoted: extracellular matrix production; proliferation and osteogenic differentiation; cell attachment, migration, and survival
Arginine-glycine-aspartic acid (RGD) [^389−392^]	3	Integrin binding sites	Upregulated: ALP, RUNX2, osteocalcin, osteopontin, BSP, Sox9, Aggrecan, fibronectin, and collagen II	Promoted: Proliferation, mineralization, and osteogenic differentiation; cell attachment and survival
Ser-Val-Val-Tyr-Gly-Leu-Arg (SVVYGLR) [^393−395^]	7	RGD binding sites	Upregulated: integrin avb3	Promoted: proliferation and neovascularization; angiogenesis and osteogenesis; adhesion, migration; tube formation of endothelial cells
			Suppressed: NFAT, osteoclastogenesis-related mRNAs	
Glycine-phenylalanine-hydroxyproline-arginine (GFOGER) [^396−399^]	4	integrin a2b1 binding sites	Upregulated: integrin a2b1 binding	Promoted: differentiation, bone regeneration, and osseointegration
Collagen binding motif (CBM) [^400, 401^]	28	Collagen binding sites	Induced sustained activation of ERK; induced transactivation of SRE, CRE, and AP-1; induced expression of type X collagen	Promoted: bone-related cell adhesion and growth; osteogenic differentiation
Fibronectin-derived peptides (FN-derived peptides) [^402−404^]	7	–	–	Promoted: bone-related cell spreading; adhesion and mineralization
**BMP-Derived Peptides**				
P17-BMP-2 [^405^]	17	–	–	Promoted: bone repair; osteoblast differentiation and bone regeneration
P20-BMP-2 and P24-BMP-2 [^406^]	20 and 24, respectively	–	Upregulated: OCN, Runx2, and collagen I	Promoted: osteogenesis and differentiation of MSCs into osteoblasts
BMP-7-derived peptide BFP-1 [^407^]	15	–	Upregulated: expression of CD44, CD47 and CD51	Enhanced: Ca2p content in cells; ALP activity; bone regeneration
**Other Peptides**				
Calcitonin gene-related peptides (CGRPs) [^61, 408−410^]	37	Pathways: cAMP, Wnt, and AMPK-eNOS	Upregulated: expression of IGF-1, IGF-1 receptor and BMP-2 receptor; ALP, OC, and COLLA1	Promoted: cell proliferation, osteogenic differentiation, and angiogenesis
				Downregulated: apoptosis and inflammation
Parathyroid hormone (1-34) (PTH 1-34) [^411, 412^]	34	Pathways: G(q)-signaling, b-arrestin recruitment, ERK 1/2 phosphorylation and phospholipase C	Upregulated: expression of Runx2 and COL2A1	Promoted: cell proliferation and chondrogenesis
			Downregulated: expression of ALP and BMP-2	
Osteogenic growth peptides (OGPs) [^413−415^]	14	Pathways: G1 protein-MAPK and RhoA/ROCK	Upregulated: osteocalcin, collagen, BMP-2, ALP and mineralization TGF b1, TGF b2, TGF b3, FGF-2, IGF-I	Promoted: cell proliferation and osteogenic differentiation; cartilage-to-bone transition
				Downregulated: adipogenic differentiation
Thrombin peptide 508 (TP508) [^389, 416^]	23	Pathways: JAK/STAT, NF-κB, PDGF, PI3K/AKT, PTEN, and ERK/MAPK; cell cycle-G1/S checkpoint	Upregulated: expression of Runx2 and OPN	Promoted: cell proliferation and osteogenic differentiation; chemotaxis, angiogenesis and revascularization
				Downregulated: apoptosis, the effect of hypoxia
NEMO-binding domain peptide (NBD) [^417, 418^]	6	Pathway: NF-κB	Downregulated: TRAP activity, actin rings; RANKL-induced c-Src kinase activity	Promoted: osteogenic differentiation of cells
				Downregulated: bone resorption
Cell-penetrating peptide (CPP) [^419, 420^]	30	–	–	Transcription factor delivery of bone regeneration-related proteins or factors into cells
AcN-RADARADARAD-ARADA-CONH_2_ (RADA16-I 16) [^421^]	16	–	Upregulated: expression of Runx2 genes, ALP, and osteocalcin	Transcription factor delivery of bone-regeneration-related proteins or factors into cells

Another group, the BMP-derived peptides (BMPs), are mostly GFs, which are responsible for inducing the formation of bone or cartilage.^34^ BMPs that promote the bone healing response are mainly derived from BMP-2, BMP-7, and BMP-9. Studies have shown that BMP-derived peptides induce the osteogenic differentiation of hMSCs and bone regeneration. Moreover, BFP-1 enhanced the Ca 2p content in cells and induced their alkaline phosphatase (ALP) activity.^35^ Selected examples of BMP-derived peptides that have been used for bone repair and regeneration studies are shown in Table 1.

In addition to ECM- and BMP-derived peptides, other peptides like calcitonin gene-related peptide (CGRP), parathyroid hormone, osteogenic growth peptides, or cell-penetrating peptides have also been studied concerning their potential to induce bone regeneration (Table 1).^34^

Traditional bone graft can be substituted with injectable self-healing hydrogel loaded with peptides: osteogenic KP and angiogenic QK, which were designed from BMP2 and VEGF, respectively, to improve osteogenic differentiation and vascularization. Both peptides: KP and QK seemed to act synergistically by promoting bone formation in rat calvaria.^36^

Although biomaterials support healing processes, their modification with peptide sequences can improve antimicrobial, proangiogenic, and immunomodulatory properties. New peptides with biofunctional activities are being discovered.^37^ Thus, various scientific groups^38−41^ employed genomics to identify new short peptides, indicating their immunomodulatory properties toward keratinocytes, periodontal ligament cells, or endothelial cells in the context of regeneration, cytokine secretion, cell apoptosis, or viability. Also, peptides are frequently incorporated into biomaterials to improve the repairing of cardiovascular tissue.^37^

### Amino Acids

Amino acids are the building blocks of proteins. Polar and charged amino acids (AAs) are abundant in NCPs and involved in bone HA mineralization. The acidic domains of NCPs (e.g., OPN, bone sialoprotein, dentin matrix protein 1, and dentin phosphophoryn) are rich in negatively charged AAs, such as aspartic acid (Asp), glutamic acid (Glu), and phosphoserine (PSer). Such negatively charged AAs play a critical role in controlling HA nucleation and growth, and they also take part in bone and dentine HA mineralization. Positively charged AAs, such as arginine (Arg) and lysine (Lys), are involved in HA nucleation within ECM proteins such as collagen.^42,43^ Moreover, Arg and Lys may accelerate the process of bone fracture healing by improving collagen synthesis and local blood supply and supplementing GFs. In addition, Glu, Arg, and Lys boost bone mineral density (BMD) by stimulating growth hormone (GH) production.^44^

Since amino acids containing amino groups can be used as aminolysis agents for biomaterials, three amino acids such as Ser, Gly, and Lys can be used to modify PLLA by surface modification to obtain nanofiber scaffolds. As shown by Zhang et al.,^45^ a modification of PLLA nanofiber scaffolds with Ser, Gly, and Lys helped to improve the hydrophilic properties of such biomaterials, as well as to lower the pressure resistance of modified scaffolds.

### GFs

The role of GFs has been widely recognized in the bone repair process. GFs are released by cells in the inflamed area. Those polypeptides regulate the events that occur during wound healing.^46,47^ The term growth factors refers to a class of polypeptide hormones that stimulate a wide range of cellular events, such as cell proliferation, chemotaxis differentiation, and ECM protein production. GFs can act locally and systematically to stimulate cell growth and function in several ways. Their activity is mainly regulated by binding to ECM receptors. Tissue repair animal model studies have provided evidence that GFs, such as epidermal growth factor (EGF), transforming growth factor (TGF)-α, TGF-β, platelet-derived growth factor, and fibroblast growth factor (FGF), are the key agents involved in the wound healing process. More importantly, studies have shown that a lack of any of these mediators at the injured site hampers the healing process. Thus, exogenous GFs are considered potent supplements in wound healing, serving as the foundation for upcoming regenerative therapies.^48^

One of the families of growth factors that have been well-studied in bone regeneration is the family of BMPs. These proteins belong to the TGF-β superfamily and have been extensively studied in preclinical and clinical investigations of bone regeneration, including bone defects and spinal fusion. BMPs have been shown to be closely related to the processes of bone formation and regeneration.^49^ In the human genome, 20 genes encode functional BMPs.^50^ Bone regeneration is, in part, a recapitulation of embryonic development. Key steps during bone morphogenesis are progenitors/stem cell chemotaxis and their proliferation and differentiation. The mechanism of action of BMPs involves signaling in all of these steps (chemotaxis, proliferation, and differentiation of osteoprogenitor cells) and, thus, the induction of bone formation by these cells. Thus, recombinant BMPs 2 and 7 have been approved by the Food and Drug Administration (FDA) for spine fusion, fracture healing, and oral surgery.^34,49^

FGF2, or basic FGF (bFGF), is the most common FGF used in regenerative medicine, including bone regeneration,^51^ and its levels are increased in acute wounds. FGF2 plays a role in granulation tissue formation, re-epithelialization, and tissue remodeling. It may also regulate the synthesis and deposition of various ECM components, increase keratinocyte mobility during re-epithelialization, promote fibroblast migration, and stimulate collagenase production.^52^ In addition, FGF2 was shown to promote angiogenesis.^53^

One of the most essential parts of the fracture healing/bone regeneration process is the state of the local vasculature. Thus, VEGF substantially stimulates local vascular regeneration in the fracture area. It has been shown that VEGF can increase MSC chemotaxis and stimulate osteoblast differentiation and proliferation. Therefore, VEGF plays a crucial role in new bone formation. *In vitro* studies have reported that VEGF stimulates the growth of vascular endothelial cells, which are the basic units of arteries, veins, and lymphatic systems. Notably, angiogenesis plays a critical role in endochondral ossification and, thus, the transformation of avascular cartilage tissue into vascular bone tissue. VEGF is released during this process by hypertrophic chondrocytes and causes the ingrowth of metaphyseal blood vessels through cartilage tissue and the formation of new bone.^54^

Chen and Wu et al.^55^ showed that applying stromal-derived factor-1α (SDF-1α) and TGF-β1 to damaged cartilage can promote the migration and chondrogenic differentiation of MSCs. SDF-1α is a chemokine and the ligand of C–X–C chemokine receptor type 4 (CXCR-4) that induces stem cell recruitment and migration. TGF-β1 is a critical regulator of the chondrogenic differentiation of MSCs. Studies have reported that combining SDF-1α and TGF-β1 has a synergistic effect on enhancing *in vitro* chondrogenic potential and *in vivo* cartilage regeneration.^55^

### Hormones (Cofactors)

The proper functioning of the endocrine system sustains skeleton development. Hormones are signaling molecules that act distal to their production site (the endocrine effect). They also regulate the synthesis and action of local factors, which directly affect cellular metabolism (autocrine and paracrine effects). Among the most critical hormones in bone formation-related processes are thyroid hormones, parathyroid hormone (PTH), calcitonin, calcitriol, androgens, estrogens, progesterone, insulin, glucocorticoids, and GH;^13^ and among these, the most important are GH and calciotropic hormones (PTH, calcitonin, and metabolites of vitamin D).

Thyroid hormones have opposite effects on bone. They stimulate the synthesis and mineralization of the osteoid matrix by osteoblasts and stimulate resorption by increasing the number and function of osteoclasts. The clinical outcome of the latter effect is bone loss in hyperthyroidism.^56^

Calcium homeostasis is controlled by PTH through its direct actions on the bone and the kidneys and indirect actions on the intestine.^57^ PTH is a signaling molecule shown to have the potential to enhance bone regeneration in significant bone defects. The potential of PTH lies in its anabolic effect on bone. The FDA has approved a treatment for osteoporosis that encompasses daily injections of PTH, which increases BMD and bone volume. Therefore, PTH may promote bone regeneration and be an alternative to autografts and BMPs to treat large segmental defects and nonunions.^58^ In a human case study documenting treatment with internal fixation, external fixation, and autograft combined with BMP-7 administration, the nonunion persisted unless the patient was supplemented with PTH 1-84.^59^

Calcitonin is an inhibitor of bone resorption that reduces the number and activity of osteoclasts. Nonetheless, calcitonin appears to have only a transient effect, as osteoclasts seem to become nonresponsive to calcitonin within a few days.^60^*In vivo* studies have shown that CGRP also plays a role in bone development, metabolism, and repair. CGRP is a 37 residue peptide generated in specific neurons by alternative splicing of the calcitonin gene. *In vitro* studies have demonstrated that CGRP may stimulate osteoblast proliferation, differentiation, and maturation in osteoblast cell lines and bone marrow MSCs.^61^

Calcitriol is a steroid hormone that promotes bone mineralization. It increases the intestinal absorption of calcium and phosphate; thus, its activity is beneficial for the growth of the skeleton.^60,62^

Sex hormones can also affect bone in numerous ways. Among others, androgens have an anabolic effect on bone by stimulating osteoblast receptors. Androgen deficiency has been associated with lower BMD, and testosterone administration to younger individuals was found to increase overall bone mass. Consistent with these findings, women with excess androgens also have higher bone densities than women with low/average levels of these hormones. Estrogens have a dual effect on bone metabolism. They favor bone formation by increasing the number and improving the function of osteoblasts; however, they also reduce resorption. Studies have shown that estrogens can increase the level of osteoprotegerin (OPG), which inhibits resorption. Thus, estrogens may play an essential role in the regulation of osteoclastogenesis. Moreover, progesterone has an anabolic effect on bone tissue. This effect may be direct, through the osteoblasts that possess hormone receptors, or indirect, through competition for the osteoblastic receptors of glucocorticoids.^60,63^ Scientific evidence has shown that high doses of glucocorticoids may have a catabolic effect on bone. This effect may be due to the inhibition of insulin-like growth factor (IGF-I) synthesis by osteoblasts and direct suppression of BMP-2 and Cbfa1, critical factors in osteoblastogenesis. In contrast, it has been demonstrated that glucocorticoids have an osteogenic capacity at physiological doses that promotes osteoblastic differentiation.^64^

Another hormone that might be involved in the bone regeneration process is insulin. It has been proposed that insulin could stimulate osteoblast differentiation, which would enhance the production of OC, and subsequently, OC may be able to stimulate pancreatic β cell proliferation and skeletal muscle insulin sensitivity. It is still uncertain whether insulin stimulates bone directly or indirectly by increasing muscle work and, therefore, skeletal loading.^65,66^

### Flavonoids

New strategies are constantly being developed to promote the natural healing of bone lesions or regeneration. Medicinal plants are essential sources of compounds such as phytochemicals, vitamins, and other nutrients, and such compounds derived from plants may enhance bone healing. Phytochemicals, especially flavonoids, may improve bone health due to their antioxidant and anti-inflammatory properties. Moreover, due to their inhibition of osteoclast cells and increased proliferation of osteoblasts, these compounds might help prevent bone loss and reduce inflammatory processes without producing the undesirable side effects of allopathic drugs.^67^

Flavonoids can be divided into various classes based on their chemical structure.^68^ Recent reports have shown that the flavonols quercetin and kaempferol can reduce bone resorption *in vitro* by directly targeting mature osteoclasts via the estrogen receptor (ER). Quercetin has anti-inflammatory properties and has been found to inhibit the proliferation of human adipose tissue-derived stromal cells and promote their differentiation into osteoblasts. Thus, quercetin can promote osteoblast differentiation and inhibit osteoclastogenesis, so it might be considered a potential drug for bone diseases and regeneration.^69^

In traditional Chinese medicine, another flavonoid, naringin, is commonly used to treat osteoporosis and bone disorders. Studies have shown that naringin may promote the proliferation of bone marrow stromal cells (BMSCs), enhance the levels of BMPs, and increase the expression of bone markers (ALP, OCN, and OPN). It has also been demonstrated that naringin can abolish osteoclastogenesis and bone resorption by inhibiting RANKL-induced NF-κB and ERK activation^70−74^

Hesperidin, whose effect on bone metabolism has been studied in rats, has been shown to improve femoral strength in adult rats and the total metaphyseal and diaphyseal BMD at the femur in young rats. However, poncirin (a flavanone glycoside) enhances the gene expression of the osteogenic transcription factor Runx2 and a transcriptional coactivator with a PDZ-binding motif (TAZ) and upregulates the expression of bone markers such as ALP and OCN in C3H10T1/2 cells. Hesperidin also promotes bone mineral deposition in BMSCs.^69^

Silymarin (Smn) is another active polyphenolic flavonoid that has been used primarily due to its antioxidant and anti-inflammatory properties. By regulating the bone formation, Smn has been shown to be effective in treating bone fractures and osteoporosis. In *in vitro* and *in vivo* models, Smn directly affected cell adhesion, proliferation, and matrix secretion and the expression of osteogenic markers such as Col I, OCN, and Runx2. Notably, an enhanced regenerative process that provides more significant bone matrix deposition and tissue organization has been observed in *in vivo* models testing the activity of Smn.^73^

### Plant Sterols

It has been suggested that phytosterols may affect osteoblast proliferation and differentiation. *Cissus quadrangularis* (*Vitaceae* family, plant kingdom) is a plant species indigenous to southern Asia and Africa that has been widely studied in bone regeneration research.^74^*Cissus quadrangularis* extract (CQE) contains steroids that are considered positive stimulants of osteoblasts and bone growth and is used as a composite modification designed for bone healing. To date, alginate *O*-carboxymethyl chitosan (O-CMC) or poly(ε-caprolactone) PCL/HA composites have been modified with CQE to study their effect on osteoblasts. Composites with CQE cause a significant increase in peptide absorption; peptides are absorbed by the composite due to the electrostatic interactions between the protein and composite surface. Cellular research indicates that these biomaterials enhance cell attachment to the composite surface and cell spreading throughout the composite. Cell proliferation increased significantly after only 72 h of stimulation, but it was suggested that CQE further enhances cell proliferation as the contact time increases. ALP, a marker of osteoblast differentiation, was significantly increased compared to that in the unmodified composite, and the effect grew over time. Moreover, the increase in ALP over time correlates with the significant increase in biomineralization by osteoblasts in the presence of a composite containing CQE compared to an unmodified composite (hydroxyapatite was detected by chemical analysis).^75−77^ However, the mechanism of CQE has yet to be determined, and more research is needed.

Seaweeds are marine plants that are widely present in Asian diets. Seaweeds have been studied for several years due to their bioactivity and potential use as pharmaceutical agents. One compound found in seaweed that has been studied is fucosterol, which is thought to affect bone regeneration.^78^ Studies have investigated the use of fucosterol in osteoblast cell culture and ovariectomized female rats (an animal model of osteoporosis). Interestingly, the obtained results indicated that fucosterol increased ALP activity, mineralization, and bone density and significantly increased bone cell proliferation. On the other hand, it was suggested that fucosterol might decrease osteoclast differentiation and affect bone resorption, maintaining bone homeostasis, which is the balance between bone mineralization and resorption. Fucosterol can also enhance the production of OC and the reduction in CTx. Moreover, the effect of fucosterol was compared to that of estradiol, which has been presented as a postmenopausal osteoporosis therapy factor, and in many cases, fucosterol was superior.^79,80^

Studies have shown that phytohormones may play a role in bone regeneration. β-Ecdysterone is a steroid hormone found in plants such as *Achyranthe bidentata*. β-Ecdysterone-mediated stimulation of osteoblasts results in significantly increased ALP levels and OPN activity. Moreover, β-ecdysterone may enhance mineralization and bone tissue formation *in vitro*. Gene sequencing analysis showed that genes involved in the BMP pathway were upregulated by β-ecdysterone. *In vivo* studies on the effect of β-ecdysterone on bone regeneration were performed using rat femurs. Four and eight weeks after bone defect initiation and β-ecdysterone injection, micro-CT imaging showed changes in the bone that were typical of healing; moreover, the bone density had significantly increased. Finally, a significant increase in the level of BMP-2 expression was detected, and this result was confirmed by an immunohistochemistry assay.^81^

### Oxysterols

Oxysterols are small, cholesterol-derived molecules naturally occurring in human and animal tissues and blood circulation that have been reported to be osteoinductive factors.^82^

20*S*-Hydroxycholesterol and 22*S*-hydroxycholesterol are compounds formed during the oxidation of cholesterol. Studies indicate that these compounds may affect the differentiation of osteogenic cells both *in vitro* and *in vivo*.^83^ In the context of alveolar bone regeneration, oxysterols were shown *in vitro* and *in vivo* to significantly enhance ALP activity, mineralization, and calcium ion levels needed for proper regeneration. Oxysterols also promote increased osteogenic gene and protein (OCN or Runx2) expression. In addition, oxysterols stimulate an increase in Hedgehog pathway activation in which proteins such as Smo (a Hh receptor) or Gli1 (a transcription factor) are involved. *In vivo* studies performed on rats showed progressive bone formation 10 and 15 days after extraction using micro-CT imaging and histological analysis; however, immunohistochemical analysis showed increased expression of ALP and OCN. In these studies, the promotion by oxysterols was at a level comparable to that of BMP-2.^84^ The above-mentioned studies align with the *in vitro* research performed by Kwon, Lee, Hwang, and Heo.^85^ Additionally, Aghaloo et al.^86^ performed *in vivo* studies on rats with poly(lactic-*co*-glycolic acid) (PLGA) scaffolds coated with oxysterols, and Johnson et al.^87^ performed studies on rats with collagen sponges containing various types of oxysterols (Oxy34 and Oxy49). All of the above-mentioned studies indicated that treatment prompted increased factors involved in bone regeneration.

Oxy49 is an oxysterol examined as a potential factor that can promote bone regeneration. *In vivo* studies performed using a rabbit cranial bone defect model and a collagen sponge containing Oxy49 showed increased expression of the osteogenesis markers COL1, OSX, OPN, and OCN. Additionally, the activity of ALP, the level of OC, and the mineralization process significantly increased. Finally, micro-CT analysis showed precise bone regeneration and density intensification after a collagen sponge containing Oxy49 was implanted into the cranium.^82^

Oxysterols are still being examined as relatively new compounds in bone regeneration. In addition to 20*S*-hydroxycholesterol, 22*S*-hydroxycholesterol, Oxy34, and Oxy49, studies on oxysterols have also included Oxy4, Oxy 18, and Oxy21, and all of these compounds may successfully promote osteogenesis. Notably, the potential of oxysterols is comparable to or even better than that of BMP-2.^88^

### Liposomes

Liposomes are lipid-based biocompatible vesicles widely used in therapies for bone healing to deliver drugs/bioactive particles and act as stimuli-responsive factors. Scaffolds containing liposomes have been proven to enhance bone regeneration. They help with the delivered molecule’s solubilization, bioactive stabilization, or bioavailability. Liposomes combined with factors that promote bone healing enhance osteogenesis. Liposomes can deliver oxysterols, and the combination of these two factors enhances osteoregenerative processes both *in vitro* and *in vivo*.^89^ Recently, novel liposomal nanocarriers, stereosomes, were developed and examined as agents to improve molecular stability. Lee et al.^90^ produced and studied stereosomes containing 20*S*-hydroxycholesterol and purmorphamine coated on PLGA and polydiacetylene (PDA) layers that can activate bone regeneration by enhancing the Hedgehog signaling pathway, which is crucial for effective osteogenesis. Applying this stereosome resulted in a synergistic increase in ALP activity and level of mineralization in cells. Moreover, the studied biomolecules caused significant increases in the expression levels of genes involved in osteogenesis (ALP, Runx2, OCN, OPN, Col1, and Gli1). *In vivo* research performed on mice confirmed the cell study results, and micro-CT and histological analysis showed an increase in bone regeneration and mineralization in stereosome-treated animals compared to that in controls. Immunohistochemical analysis indicated enhanced expression of the osteogenic markers Runx2 and OCN. Research by Lee and colleagues^90^ is in-line with that of Cui et al.^83^ on stereosomes containing 20*S*-hydroxycholesterol and sterylamine.

Liposomes were formerly studied as effective agents to deliver the bone morphogenetic protein BMP-2 gene to the bone fracture site, which resulted in enhanced bone regeneration.^91^ Currently, liposomes are successfully being used both as individual carriers of biomolecules and as additions to scaffolds.^92^

### Statins

Statins are well-known drugs used to lower LDL cholesterol levels and prevent the development of atherosclerosis. Almost 20 years ago, it was reported that hypercholesterolemic patients undergoing statin therapy had a reduced risk of bone fracture. Researchers have thus started investigating how BMD and turnover change after statin therapy and how statins may affect bone regeneration. Montagnani et al.^93^ examined 30 women suffering from postmenopausal hypercholesterolemia. The studied group was treated with simvastatin daily for 1 year. During that time, the group did not receive any treatment that would affect bone metabolism (calcitonin, calcium, and vitamin D). Blood samples were collected every 3 months, and serum calcium, phosphate, and ALP levels were measured. Moreover, bone resorption and mineral density were assessed. The obtained results indicated that the treated patients had significant increases in total and bone ALP levels over time and BMD in the lumbar spine and femoral neck. In the same year, Ayukawa, Okamura, and Koyano^94^ performed a study on rats in which titanium implants were installed in the tibias, and a daily dose of simvastatin was given. The bone contact ratio and bone density measurements showed significant increases in the experimental group compared to that in the control group, which was not treated with simvastatin. Histological analysis showed newly formed bone and abundant bone trabeculae in the treated animals. Wong and Rabie^95^ investigated whether adding statins accelerates osteogenesis in rabbits. After implantation of a collagen sponge combined with simvastatin into the calvarial fracture, the expression levels of VEGF, BMP-2, and Cbfa1 were enhanced and resulted in earlier osteoinduction and neovascularization. Wong and Rabie^96^ also performed histological analysis to identify new bone formation that occurred 5 days after implantation of a simvastatin-modified collagen sponge.

Importantly, simvastatin is not the only statin compound studied in the context of bone regeneration. Moriyama et al.^97^ investigated whether local fluvastatin application promotes osteogenesis after PLGA implantation into rat tibiae. Tibias were used for histological analysis 1, 2, and 4 weeks after implantation, indicating a significant amount of osteoid bone and increased mineralization. Masuzaki et al.^98^ showed by histological analysis that, after fluvastatin-modified PLGA microsphere implantation into rat tibiae, bone formation was amplified, and the bone implant contact significantly increased. Additionally, the level of OCN, a bone metabolism marker, was significantly higher 2 and 4 weeks after implantation. Research by Rakhmatia, Ayukawa, Furuhashi, and Koyano^99^ aligns with previous studies. Rats implanted with fluvastatin-modified carbonate apatite showed enhanced bone formation and bone volume by micro-CT analysis. Moreover, histological analysis confirmed these results and indicated significant intensification of bone mineralization.

In addition, *in vitro* research indicated that statins regulate the OPG/RANKL/RANK pathway. Statins can inhibit bone resorption, ROS generation, or osteoclastogenesis. Additionally, statins may affect osteogenesis promoters, such as BMP-2, TGF-β, or ALP. Statin-stimulated cells exhibited increased expression of the osteogenic genes Runx2 and OCN and the osteogenic proteins Runx2, OCN, and OPN.^100^

### Microbial Biopolymers

Bacteria and microscopic fungi produce natural polymers as part of their intrinsic physiology to create a mechanical protective layer that surrounds their cells. These polymers store molecules necessary for proper metabolism functions and create a biofilm that protects their cells from the harmful effects of the environment. Microorganisms can synthesize various types of biopolymers with different monomer compositions, molecular weights, 3D configurations, and cross-linking arrangements that can be tailored for specific applications in BTE.^101^ Microbial polymers are synthesized from enzymatic reactions that link monomers, such as sugars, amino acids, or hydroxy fatty acids, to create high molecular weight molecules. Microorganisms can produce various classes of biopolymers with potential biomedical applications, such as polysaccharides, polyamides, polyesters, and polyphosphates.^102^

Bacterial cellulose (BC) is a linear homopolysaccharide biopolymer produced by many Gram-negative bacterial genera, such as *Komagataeibacter* (formerly *Gluconacetobacter*), *Agrobacterium*, *Acetobacter*, *Burkholderia*, *Erwinia*, *Pseudomonas*, and *Rhizobium*.^103,104^ BC is synthesized from glucose in the periplasmic space of bacterial cells by cellulose synthase, and its chemical structure is composed of β-d-glucopyranose units linked by β-1,4 glycosidic bonds. The biocompatibility, biodegradability, high crystallinity, porosity, and tensile strength with mechanical robustness make BC an interesting biopolymer that can be used in designing modern biomaterials for the targeted regeneration of bone tissue.^103^

Bassi et al.^105^ showed that intracranial implantation of a BC membrane led to bone neoformation and vascularization at the defect site and confirmed the activity of key ossification markers such as OC and OPN 60 days after biomaterial implantation.^105^ BC is used by itself and in combination with other bioactive factors in designing biomaterials for bone regeneration. Hydrogels made from BC modified with gold nanoparticles significantly increased the activity of ALP, OC, and OPN in cell culture models and led to the formation of apatite deposits. In contrast, in a rabbit model, these hydrogels showed that new bone tissue with high mineral density had been formed.^106^ Similar conclusions were drawn by Kheiry et al.,^107^ who showed that modifying BC with fisetin contributes to the increase in ALP activity and the concentrations of OC and OPN in mesenchymal cells subjected to osteogenic differentiation.^107^ Nanocomposites of BC modified with hydroxyapatite (HA), the main inorganic compound responsible for the mechanical properties of bones, promoted the proliferation and maturation of mesenchymal cells into osteocyte precursors and effectively contributed to the neoformation of bone tissue after implantation.^108^ Unmodified BC does not have antibacterial properties; however, its porosity and good ability to biofunctionalize with molecules such as antibiotics, silver nanoparticles, lysozyme, or cationic surfactants can be used to design biomaterials that reduce the risk of postimplantation infections.^109,110^

Another example of microbially produced polymers with potential for use in bone regeneration are β-glucans. β-Glucans are heterogeneous groups of polysaccharide polymers composed of d-glucose monomers linked by (1 → 3), (1 → 4), or (1 → 6) glycosidic bonds. The cell walls of grains, bacteria, fungi, and yeast are a natural source of this biopolymer. The most well-known β-glucans synthesized by microbes are the linear (1 → 3) and branched (1 → 3;1 → 6) β-glucans found in *Saccharomyces cerevisiae*. The physiochemical and biological properties of β-glucans strongly depend on the source, extraction method, polymer chain length, and extent of purification.^111,112^

One of the fundamental problems in achieving an appropriate level of osseointegration with an implant is the excessive bone resorptive activity of osteoclasts. There is substantial scientific evidence to conclude that polycan, a β-glucan derived from *Aureobasidium pullulans*, reduces the number of active osteoclasts and inhibits the secretion of pro-osteolytic cytokines such as interleukin-1β (IL-1β) and tumor necrosis factor-α (TNF-α). β-Glucan, which is part of the *S. cerevisiae* cell wall, contributed to the downregulation of receptor activator for nuclear factor κB ligand (RANKL) and the upregulation of OPG, which resulted in the inhibition of bone loss in a mouse model.^113^ The suppressive activity of β-glucan derived from baker’s yeast against RANKL has also been demonstrated by Hara et al.^114^ Stimulation of mouse bone marrow cells with *S. cerevisiae* β-glucan inhibited differentiation from maturing osteoclasts by downregulating the nuclear factor of activated T cells 1 (NFATC1), which was caused by the suppression of NF-κB signaling and c-fos expression.^114^ β-Glucan can not only inhibit osteoclast activity but also be used as a polymer when designing biocomposites modified with a ceramic phase. Biocomposites composed of (1 → 3) β-glucan and HA meet important physicochemical requirements, such as the ability to undergo thermal sterilization without damaging the polymer structure, good porosity, flexibility, and self-adaption to the defect shape.^115^ Modifying such composites by adding HA-containing carbonate ions (CHA) increased the solubility and decreased the crystallinity of the ceramic phase, as well as intensified the attachment, proliferation, and differentiation of osteoblasts. In rabbit models, 6 months after implantation, the CHA/β-glucan composite contributed to the increased formation of new cortical bone and intensified mineralization at the implantation site.^116^

An example of a linear bacterial (1 → 3) β-glucan that has aroused interest in the design of new biocomposites for targeted bone tissue regeneration is Curdlan, which is produced by *Alcaligenes faecalis*.^117^ Curdlan limited osteoclast differentiation by suppressing NFATC1 activation via downregulation of the Syk kinase signaling pathway, which is responsible for osteoclast differentiation, maturation, and bone lytic activity.^113,118^ Curdlan can be modified to make highly elastic and biocompatible hydrogels or biocomposites. Curdlan/whey protein isolate/hydroxyapatite biomaterials showed a high cytocompatibility level and promoted OC production in an in vitro model of human osteoblasts.^119^ The addition of Curdlan to a chitosan/HA scaffold improved the porosity, water uptake capability, and biocompatibility of the composite and enhanced human osteoblast survival and proliferation on the scaffold, which are crucial to start the implant osseointegration process.^120^ Toullec et al.^121^ reported that Curdlan–chitosan scaffolds were not cytotoxic and improved cell migration on the surface of the biocomposite; however, further studies are required to demonstrate the positive effect of this biomaterial on bone tissue regeneration.^121^

Bacterial exopolysaccharides (BEPSs), such as gellan and alginate, are classified as high molecular weight carbohydrate polymers and are secreted by cells into the external environment. BEPSs perform various physiological functions and can be adapted to the needs of regenerative medicine due to their unusual physicochemical properties.^122^ Gellan isolated from *Sphingomonas paucimobilis* was incorporated into a composite in the form of a gum, and the addition of HA increased the adhesion of human adipose-derived stem cells to the surface.^123^ Alginate secreted by *Pseudomonas aeruginosa* is being studied for use in bone tissue regenerative medicine as a carrier of GFs. The delivery of BMP-2 and BMP-7 using an alginate biomaterial enhanced the differentiation of bone marrow-derived stem cells to osteoblasts, and codelivering the BMP-2 and VEGF released from the alginate gels improved the reconstruction of bone defects.^124^

Another interesting biopolymer produced by *P. aeruginosa* is pyomelanin, a black–brown pigment formed by the oxidative polymerization of homogentisic acid.^125^ The use of melanin polymers, such as pyomelanin, seems to be an economical and affordable way to improve the physicochemical and osteoinductive properties of newly designed biocomposites.^126^ Important premises indicating the need to investigate the role of pyomelanin as a modulator of bone tissue regeneration processes are the studies of Yoo et al.,^127^ who showed that melanin isolated from *Gallus gallus domesticus* promoted the *in vitro* proliferation and differentiation of osteoblastic MG-63 cells through BMP-2 signaling and inhibited osteoclast formation.^127^

## Methods to Prepare Polymeric Materials and Scaffolds for BTE

The structure of human bone is complex and capable of bearing mechanical loads and resisting deformation.^128^ Bone is also involved in multiple vital processes, including maintaining homeostasis and regulating blood pH.^129^ Taking into consideration the complexity of bone structure, materials suitable for BTE should be capable of bearing mechanical loads, biocompatible, osteoconductive (allowing cells to move along the scaffold and slowly produce new bone),^130^ osteogenic (stimulating bone growth),^130^ and osteoinductive (stimulating stem cells to differentiate toward osteoblasts).^130^ A novel biomimetic approach to designing a biodegradable scaffold that propagates osteoconductivity for bone and cartilage tissue applications includes replicating the ECM^131^ and providing suitable conditions for tissue regeneration.

There is a diverse range of materials that are applicable for BTE. These materials include polymeric materials, bioceramics, and preferably tailored composite materials that meet the requirements for the above-mentioned properties. Currently, several methods are known for producing polymer scaffolds, polymer–ceramic scaffolds, and multicomponent materials used in BTE. Methods of producing materials for BTE can be divided into two main groups: those obtained by solvent techniques and those obtained by techniques involving plasticization of the polymer material. This paper considers the most important and popular techniques for manufacturing three-dimensional scaffolds with potential applications in BTE, emphasizing polymer and composite scaffolds.

### Polymers, Inorganic Fillers, and Composites

Polymeric materials are promising structural materials for scaffold preparation in BTE and usually act as a composite matrix and an active compound carrier (at least two ingredients). These macromolecules can be divided into those that are naturally derived and those that are synthetic. The former group includes polysaccharides, such as alginate,^129^ chitosan^129,132^ and hyaluronic acid,^133,134^ protein-based collagen,^135,136^ and gelatin,^132,137^ which are capable of forming hydrogels as well as a variety of cellulose-based biofibers.^138^

The application of natural polymers in bone regeneration systems minimizes the negative immunological response resulting from their high biocompatibility.^139^ The main disadvantage of this group of materials is their low mechanical resistance, especially considering the load-bearing requirements in BTE, as concluded by Swetha et al.^140^

Synthetic polymers are more amenable to chemical modification. For example, the presence of functional groups can allow the facile binding of cellular proadhesive ligands such as arginine–glycine–aspartic acid (RGD).^141^ On the other hand, natural collagen has an RGD sequence already incorporated into its structure. Synthetic polymers generally have higher mechanical resistance than natural polymers. Significant representatives of this group in BTE include poly(l-lactic acid) (PLLA),^142,143^ poly(ε-caprolactone) (PCL),^144,145^ poly(ethylene glycol) (PEG),^146^ and the emerging polymer poly(glycerol sebacate) (PGS).^147^

The role of inorganic ceramic materials has been significant in developing BTE since the 1990s.^148,149^ Among the most important compounds are crystalline hydroxyapatite (HA), β-tricalcium phosphate (β-TCP), and amorphous bioglasses. The modern approach to using ceramics in BTE involves their stimulation of osteogenesis by releasing active ions^150^ (e.g., Ca^2+^ in the case of HA) and the ability to act as a mechanical support in the composite with a high compressive modulus.^151^ Such compounds should be resorbable over time.

HA and β-TCP are composed of calcium phosphate and therefore resemble the inorganic phase of human bone. Calcium phosphate-based substances play a vital role in biomineralization, which is essential to strengthen the osteogenic capability of the scaffold.^152,153^ Bioglasses constitute the other class of ceramics in BTE, which are materials composed of Si_2_O, Ca_2_O, and P_2_O_5_ and can enhance osteogenesis more rapidly than calcium phosphates.^154^

Composite materials are based on at least two constituents and possess properties from each phase. Basic BTE composite materials are made of a polymeric matrix and an inorganic filler. Preparing composites aims to combine the desired features of both materials.

### Pathways to Obtain BTE Composite Scaffolds

There is a broad array of techniques to manufacture and form BTE scaffolds. These techniques depend on the desired geometry, presence, and distribution of the filler, and most importantly, the chemical characteristics of the substrates. In terms of biodegradability, porosity is of vital importance. The degradation medium (e.g., water) can infiltrate scaffolds more freely if voids (pores) are present compared to water infiltration into the bulk material. Moreover, in thermosetting polymers such as PGS,^155^ the degradation time can be adjusted by altering the curing time of the polymer bulk.^156^ Porous scaffolds naturally promote osteoconductivity toward the inner layers of the scaffold. Therefore, the synthesis of porous materials is an important subject in BTE.

### Electrospinning

The most facile method to manufacture highly porous nonwoven fibers in BTE is electrospinning.^157^ This technique utilizes an electric current to deposit the polymer solution on the substrate to form a fiber. Electrospinning has attracted attention due to its ability to mimic the tissue ECM and the wide range of materials that are applicable for spinning. The components of electrospinning systems for BTE include a thermoplastic polymer solution, such as PLA or PCL, with a combination of collagen, chitosan, silk, gelatin, hydroxyapatite, or β-TCP. The significance of such systems in terms of bone regenerative medicine is comprehensively described in a review paper by Jang, Castano, and Kim.^158^

The electrospinning method can produce two-dimensional fiber networks. However, there are more effective methods for creating three-dimensional structures that can mimic the complexity of bone tissue. In bone tissue engineering, creating porous and hierarchical structures is important, which is difficult to achieve with electrospinning. Electrospinning requires optimizing many parameters, such as voltage, solution flow, and distance between the needle and collector.^159^ The need to experiment and adjust these parameters can take time and lead to trial and error. In addition, electrospinning is a manufacturing process that is not highly repeatable. The electrospinning process uses organic solvents and/or chemicals that can be potentially toxic to cells and the body.^160^ It is necessary to ensure adequate elimination of these substances to avoid negative effects on the biocompatibility and functionality of scaffolds. Fiber produced by electrospinning often has a very low density and mechanical strength compared to natural bone tissue. This can lead to poor structural stability of the scaffold and limit its use in stressed areas. Manufacturing homogeneous composite materials is also a challenge for electrospinning.

### Thermally Induced Phase Separation (TIPS)

TIPS is one of the most popular methods to obtain three-dimensional scaffolds for BTE. In this technique, the polymer needs to be homogeneously dissolved in a solvent with a high melting temperature (*T*_m_) due to the subsequent freeze-drying process (1,4-dioxane is one of the most popular solvents in TIPS with *T*_m_ = 11.8 °C^161^). Afterward, a ceramic filler such as bioglass or apatite can be introduced to the polymer solution^162^ and dispersed by stirring or ultrasonication.^163^ After a suitable solution is obtained, a freeze-drying process is performed to remove the solvent from the composite matrix and generate pores. The main limitation of this method is the thermolability of the solvent used for scaffold preparation. On the one hand, the solvent should dissolve the polymer, and on the other hand, the solvent should be easily removed from the scaffold by lyophilization.

In the TIPS technique, an additional porogen, such as NaCl^162,164^ or a sugar,^165^ can be introduced to increase pore size by more than 100 μm. The porogen can subsequently be removed (washed away) from the matrix after lyophilization (e.g., TIPS followed by salt leaching (TIPS-SL)). This method gives the possibility of obtaining highly porous scaffolds (up to 98%)^162^ with an interconnected pore morphology.^166^ The porosity and internal structure can be tuned by altering the polymer solution concentration, filler content, amount of porogen, and particle size.^167^ There are a variety of polymer/filler compositions that have been fabricated by TIPS and reported for BTE applications. For example, PLLA/β-TCP nanocomposite scaffolds^143^ or PGS-based scaffolds^168^ can be fabricated by TIPS-SL.

The TIPS (TIPS-SL) process requires the use of organic solvents, which can affect the scaffold’s biocompatibility.^169^ Some of these solvents can be toxic to cells, which can limit the use of scaffolds in the context of tissue engineering. The TIPS process can be time-consuming (at least a few days) and requires precise control of temperature, time, and other parameters. This can lead to longer production times and increased costs. Producing scaffolds with adequate porosity and interconnected pores is crucial for bone tissue regeneration.^170^ However, the TIPS process can be difficult to control in terms of the porosity. It can be difficult to achieve uniform pore sizes and shapes, which can affect the scaffold’s effectiveness in regenerating bone tissue. The use of the TIPS-SL technique only partially solves the problem, as the pore size is increased, but at the same time, a material with lower strength parameters is obtained.^171^

### Solvent-Free Techniques

#### 3D Printing (3DP)

3DP techniques consist of slicing a computer-aided design (CAD) model into layers and its subsequent manufacture. This paper will cover only the method by which BTE scaffolds can be obtained, including techniques such as selective laser sintering (SLS),^172^ fused deposition modeling (FDM)/fused filament fabrication (FFF),^173^ and stereolithography (SLA),^174^ which are based on different operation principles. However, these methods are additive manufacturing techniques, which are, among other techniques, used to prepare scaffolds or implants for bone regeneration. There are a variety of biodegradable polymer/ceramic systems for BTE that have been manufactured by means of 3DP (Table 2). The major advantage of 3DP when manufacturing scaffolds for biomedical applications is the possibility to obtain a reproducible and well-defined architecture that meets the needs of patients.

**Table 2:** Applications of the Techniques by Which BTE Scaffolds Are Prepared^a^

**Formation technique**	**Composition**	**Remarks**	**Biological activity**	**References**
Electrospinning	PLA/PGS	Mat for cardiovascular diseases	Cardiomyocyte morphology similar to that in the natural environment	(422)
	PLLA, PLLA/HA, PLLA/collagen/HA	Composites for bone tissue engineering	hFOB 1.19 cells had a higher proliferation rate and increased ALP activity in a PLLA/collagen/HA system	(423)
	PCL/PGS	The different solvents used for fiber preparation showed no cytotoxicity	Human cardiomyocytes, cytotoxicity	(424)
TIPS	PLLA/β-TCP	Interconnected, hierarchical pore structures with a high porosity and compressive modulus in comparison to pristine PLLA scaffolds	Enhanced osteoblast (MG-63 cell) proliferation, penetration, and ECM deposition	(143)
	PDLLA/45S5 bioglass	Anisotropic, bimodal pore architecture, >90% porosity	–	(425)
	PLGA/HA	Mechanical properties and water sorption enhanced by HA addition	Significantly higher rabbit MSC proliferation on the PLGA/HA scaffold in comparison to that on the pure PLGA scaffold	(426)
FFF	PDA-coated PLA scaffold	Facile route for BTE scaffold manufacturing: FDM printing + immersion coating; the PLA scaffold was more hydrophobic than the PDA-coated scaffold	PDA-coated PLA scaffolds allowed hADSC cells to adhere and grow better than the unmodified PLA scaffolds	(176)
	PCL	Indicates PCL is an important allogenic material in the field of reconstructive craniofacial surgery	Successful reconstruction of craniofacial defects regarding new bone formation	(178)
	PLA	PLA maintained a semicrystalline structure even though the polymer chains were shortened and thermal degradation profile had changed	Printed PLA scaffolds were proven to be biocompatible and allowed bone cell colonization	(427)
SLS	PCL/HA	Gradient architecture with interconnected porosity and the desired mechanical properties	Excellent biocompatibility, induction of osteochondral repair *in vivo*	(428)
	CaP/PHBV and CHAp/PLLA	Sintered scaffolds with a biodegradable osteoconductive calcium phosphate matrix; gradual decrease in mechanical properties after immersion in PBS	In SaOS-2 cell culture, CaP facilitated ALP expression on both materials; no significant difference in proliferation or ALP activity between the CHA/PLLA nanoscaffold and PLLA scaffold	(429)
	PVA	Periodic, porous architecture; PVA is vulnerable to high laser power for SLS	Successful growth and adaptation of MG-63 cells	(184)
SLA	PCL/HA	Gradient architecture with interconnected porosity and the desired mechanical properties	Excellent biocompatibility, induction of osteochondral repair *in vivo*	(428)
	CaP/PHBV and CHA/PLLA	Sintered scaffolds with osteoconductive calcium phosphate and a biodegradable matrix; gradual decrease in mechanical properties after immersion in PBS	In SaOS-2 cell culture, CaP facilitated ALP expression on both materials; no significant difference in proliferation or ALP activity between the CHA/PLLA nanoscaffold and PLLA scaffold	(429)
	PVA	Periodic, porous architecture; PVA is vulnerable to high laser power for SLS	Successful growth and adaptation of MG-63 cells	(184)
Melt mixing/extrusion	PLLA/HA	Composites were extruded and patterned using a femtosecond laser	Human osteoblasts (ATCC CRL-11372 cells) were cultured on the laser-modified surface	(430)
	PLLA/HA	Composites extruded using co-rotating twin-screw extruder and irradiated using a CO_2_ laser	Not tested	(431)
	PLLA	PLLA foil extruded using a conical single screw extruder and irradiated using a UV laser	Not tested	(432)
	PLLA/HA	Composites extruded using a co-rotating twin-screw extruder	Human adipose-derived stromal cells (hASCs)	(433)

a Abbreviations: poly(d,l-lactide), PDLLA; poly(lactic-*co*-glycolic acid), PLGA; calcium phosphate, CaP; carbonated hydroxyapatite, CHA; poly(hydroxybutyrate–cohydroxyvalerate), PHBV; phosphate-buffered saline, PBS; alkaline phosphatase, ALP; poly(vinyl alcohol), PVA.

3D printing processes are most often conducted at high temperatures (SLS, FFF/FDM) due to thermoplastic materials being processed at temperatures as high as 160–200 °C.^175^ For this reason, the introduction of bioactive particles, which are often sensitive to temperature, is difficult. The use of UV irradiation for cross-linking during the 3D printing (SLA) process degrades the polymer from which the scaffold is made.^176^

#### FDM/FFF

An operating principle of FDM/FFF is extrusion on a thermoplastic filament (usually 1.75, 2.85, or 3.0 mm in diameter) through a nozzle, followed by deposition on the printing bed. After a layer is delivered, the extruder moves upward, and the next layer is laid. The resolution of the printout is mainly affected by the extrusion rate, motor speed, and nozzle diameter. The main advantages of FDM are the simplicity of the process and high printing efficiency. On the other hand, the main limitation involves the thermoplastic characteristics of the material with the existence of a molten phase. Additionally, the process is relatively slow and has low accuracy. Moreover, complex geometries require auxiliary supports, which are removed during postprocessing. The filament for FFF is produced by means of melt extrusion.

Polymers for BTE applications include thermoplastic materials such as PCL, poly(vinyl alcohol) (PVA), and polylactides. This technique provides the possibility of introducing a ceramic phase into the blend^177^ for superior osteoconductivity. Furthermore, the infill architecture of the FDM printout affects the *in vivo* behavior of the scaffold, as the honeycomb internal structure of the FDM scaffold has been indicated to increase bone ingrowth.^178^

#### SLS

The SLS operation principle is based on layer-by-layer fusing/sintering of particles on a powder bed by heat generated from a laser beam.^179^ By means of rollers, the printing bed is coated with a preset layer of powder, which is sintered according to the CAD model. The printing bed moves downward incrementally, and the process repeats until the final printout is completed.^179^

The SLS technique is limited by the ability of the particles to absorb the wavelength of laser light as well as the laser energy density. System optics and resolution also affect the final structure and porosity of the material. Particle size, sphericity, and chemical characteristics are of vital importance for materials submitted for SLS. Usually, microspheres with a defined size (20–80 μm) for use in the SLS process are produced by emulsion solvent evaporation.^180,181^ However, one can purchase presynthesized SLS powders, such as PCL (CAPA 6501, Solvay Caprolactones, Warrington, Cheshire, UK), poly(hydroxybutyrate–cohydroxyvalerate) (PHBV) (ICI, UK), or PVA (Nippon Synthetic Chemical Industry Co. Ltd., Japan). Among the wide array of biodegradable materials for SLS, PVA is of particular interest due to its flexibility and semipermeability, which can allow oxygen and nutrient exchange, which is necessary for the cellular culture on the scaffold to thrive.^182^

The SLS process requires the material to have a low melting point and be able to form intermolecular bonds after exposure to a laser.^183^ The main advantage of using SLS scaffolds for BTE is the possibility of obtaining a porous structure that mimics the bone ECM. The overall porosity of the SLS printout can be higher than anticipated due to the formation of micropores in the scaffold.^184^ On the other hand, this technology is expensive and requires a complex modeling procedure.

#### SLA

The SLA approach in additive manufacturing utilizes ultraviolet (UV) light to trigger selective photopolymerization. The printing procedure involves submerging the printing bed in the photopolymer reservoir with subsequent layer-by-layer exposure to UV radiation in accordance with the CAD model. The SLA method is comparable to SLS; however, SLA uses a liquid prepolymer. After the layer is photocured, the printing bed slides down, and the process is repeated until the last layer is irradiated.^185^ SLA material diversity is limited by the requirements of biodegradability and lack of cytotoxicity. Materials for SLA scaffold-based tissue engineering include derivatives of PEG acrylate, PEG methacrylate, PVA, and modified polysaccharides, such as hyaluronic acid and dextran methacrylate, in addition to poly(propylene fumarate) (PPF) and PCL-based resins.^186^

For biomedical applications, the properties of SLA resins can be adjusted; for example, reducing the percentage of DEF in the PPF resin increases the viscosity of the solution and promotes cross-linking, which results in a final product with superior mechanical properties.^187^ However, a higher degree of polymer cross-linking affects the degradation rate. Lower cross-linking degree facilitates degradation. It is a vital parameter considering the resorption of biomaterial *in vivo.*

#### Melt Mixing

Melt mixing is the most important continuous method by which polymer/filler composites are obtained. One type of melt mixing is the twin-screw co-rotating extrusion (TSCE). TSCE is the most effective means to distribute filler in the polymer matrix and enables even filler distribution, even on the nanoscale.^188^ TSCE utilizes an instrument that consists of a motor, heated cylinder, screws, hopper, die, and control equipment (thermocouple, pressure sensor). Two screws are installed inside the cylinder on a shaft and rotate in the same direction. These screws are made of configurable sections (mixing and conveying sections) that can be arranged in various configurations.^189^ A preprepared material in the form of a granule or powder is dosed into the first zone of the extruder (feeding zone). Screws, rotating with speeds usually ranging from several dozen to a few hundred, plasticize, transport, and homogenize the material to the extruder head. After this process, the material in the form of a filament is allowed to cool down (using a water bath or in air) and pelletized. The advantages of this method are the fast homogenization and good dispersion of the filler. Disadvantages of this method include thermal degradation of the polymer during the process, the need for a relatively high amount of the polymer for extrusion, and large losses during processing. Extrusion is not actually a scaffolding manufacturing method, but is a preliminary method used to homogenize composite components. The material is obtained in the form of pellets or filaments and in this form is used for 3D printing.

## Methods to Deliver Bioactive Molecules

### Bone Regeneration Biomolecule Delivery Platforms and Release Strategies

**Figure 2:**
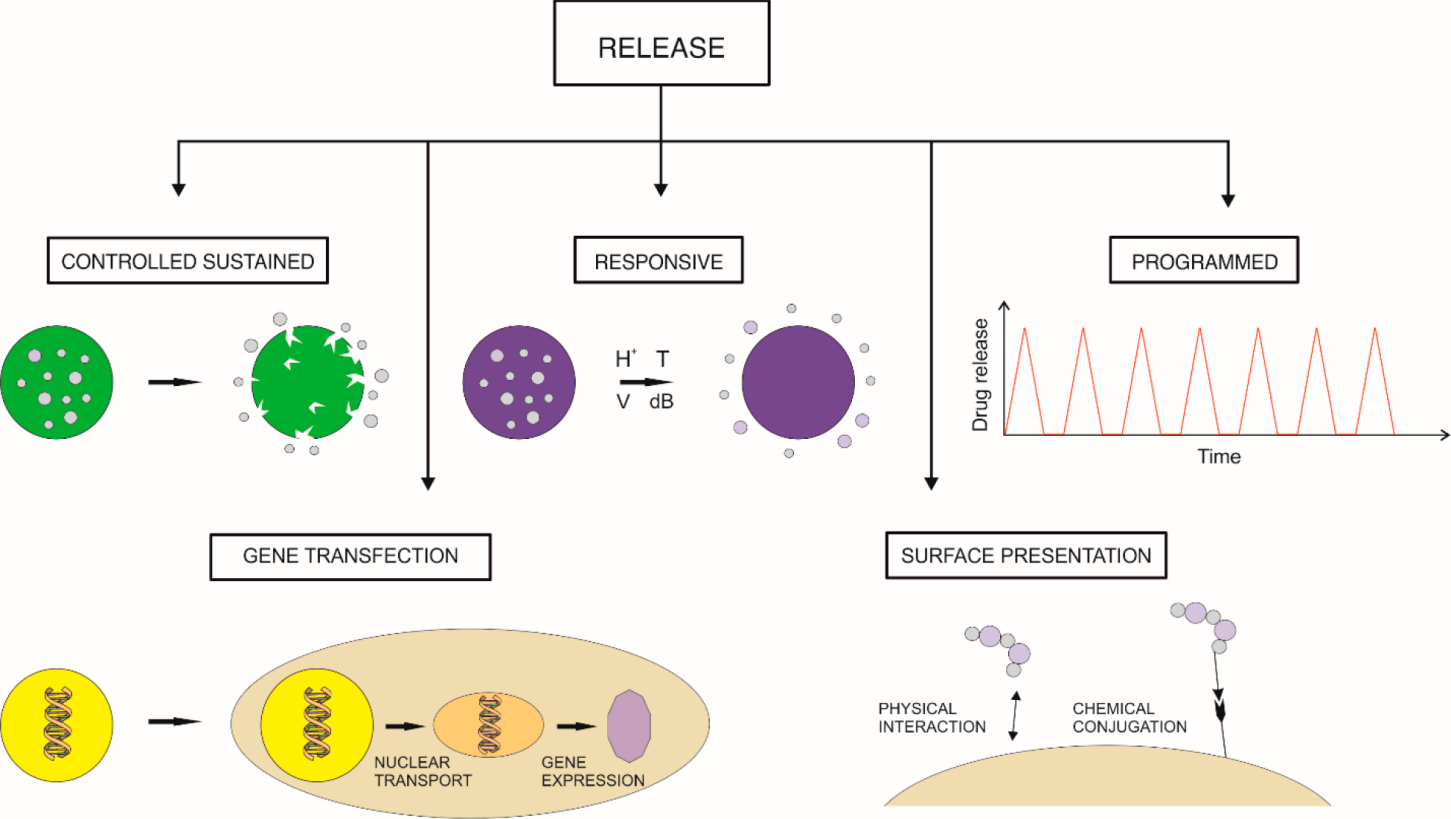
Strategies for the delivery of bioactive agents in bone regeneration.

Bone regeneration involves multiple stages, including the inflammatory phase, callus formation phase, callus removal/bone deposition phase, and bone remodeling.^8^ Each phase is driven by different biochemical signals, which have to be delivered at a specific time in a coordinated and sequential manner.^190^ To achieve the best therapeutic outcome, orthopedic implants loaded with bioactive factors should release these factors at a dose and time that reflects this physiological pattern. Biomolecule dosing should also be tailored to the patient’s clinical status, i.e., cause, location, and severity of bone defect, age, and presence of coexisting conditions. A number of biomolecule delivery platforms and release strategies have been proposed to provide treatment options customized to different types of biofactors and for different types of bone defects. The platforms developed to date provide a wide range of dosing profiles that depend on the implant material, structure and size, biomolecule immobilization technique, and amount and spatial distribution of the biomolecules. Biomolecule delivery platforms can be categorized into five main types: surface-functionalized, controlled/sustained release, preprogrammed release, stimuli-responsive, and those for gene delivery, as depicted in Figure 2.

### Surface-Functionalized Delivery Platforms

Surface-functionalized implants are being intensively explored in bone regenerative medicine^191−193^ as delivery platforms for BMPs,^194−196^ platelet-derived growth factor (PDGF),^197^ TGF-β,^198^ and vitamins D and K.^199^ During the fabrication of surface-functionalized implants, biomolecules are introduced onto the implant surface by physical adsorption,^200,201^ chemical conjugation,^191,192^ or ligand–receptor binding.

**Figure 3:**
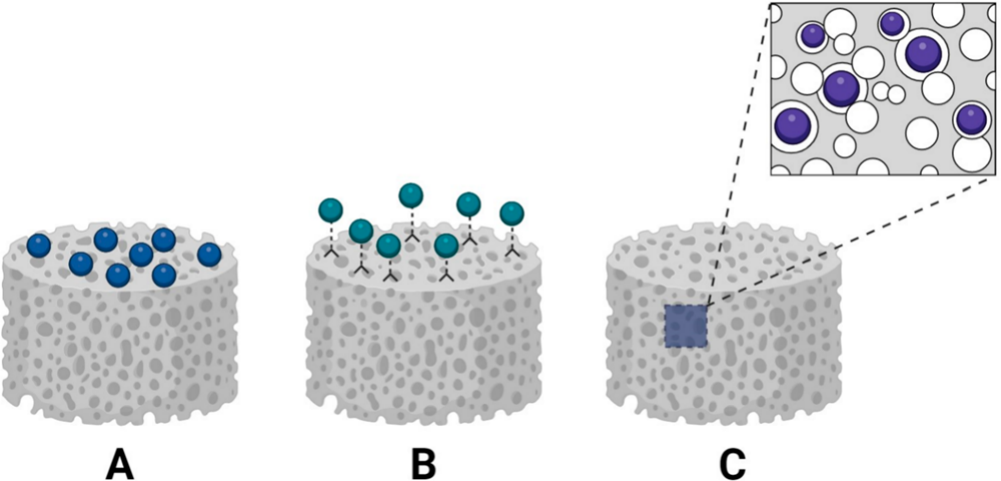
Physical and chemical strategies to immobilize bioactive compounds on biomaterials: (A) physical adsorption, (B) covalent binding, and (C) entrapment in a polymer matrix.

Physical adsorption (Figure 3A) occurs when biomolecules attach to the scaffold material via electrostatic, hydrophobic, van der Waals interactions, or hydrogen bonding.^200^ The release kinetics of biomolecules immobilized by physical adsorption depends on their affinity for the implant material and can be controlled by environmental conditions such as temperature and pH. To enhance biomolecule adsorption to biomaterials, their surface can be pretreated with charged molecules such as amino acids (e.g., serine, asparagine) or acids (e.g., pyrophosphoric acid,^202,203^ mercaptosuccinic acid^204^). Charged biomaterial surfaces can selectively attract molecules of interest (e.g., lysozyme,^205^ BMP-2^206^), while oppositely charged molecules are repulsed. Physisorption has been widely used to immobilize osteoinductive biomolecules (e.g., BMP-2,^194,195^ PDGF,^197^ TGF-β198]) in a variety of scaffolds, including collagen and gelatin sponges,^207^ poly(glycolic acid) meshes, poly(d,l-lactide) scaffolds,^208^ hydroxyapatite,^206,209^ tricalcium phosphate ceramics, and others. This technique has also been used to fabricate INFUSE Bone Graft from Medtronic (an absorbable collagen sponge soaked with BMP-2) for recombinant BMP-2 delivery, which is currently the only FDA-approved BMP-2 product that is commercially available. However, the major limitations of materials functionalized via physisorption include poor drug retention and limited control over the biomolecule release rate due to weak biomolecule bonding. This type of materials typically suffer from burst release,^210^ which is defined as a sudden initial release of a drug bolus resulting from its rapid desorption from the material surface. The main risk of burst release is the overdose of a therapeutic molecule in the immediate postimplantation period, which is usually associated with reduced drug absorption and rapid drug depletion. Supraphysiological doses of BMP-2 have also been shown to cause serious side effects such as spine swelling, neck edema, tumor formation, osteolysis, and ectopic bone formation,^211^ which remain among the biggest challenges of the current clinical approaches to bone healing based on BMPs.^211^

More stable attachment of biofactors to the scaffold surface can be achieved by chemical conjugation.^212−214^ Chemical conjugation methods are based on the formation of covalent bonds between biomolecules and biomaterials (Figure 3B) through the course of chemical reactions such as carbodiimide-mediated amidation, esterification, or click reactions.^215−217^ Since the majority of osteoinductive biomolecules are proteins, the most commonly used covalent coupling methods are based on carbodiimide-mediated reactions between the protein amine groups and carboxyl groups of the biomaterial.^193^ Due to the inert nature of many materials used in regenerative bone therapies (e.g., polyesters), these materials require surface functionalization prior to biomolecule attachment. Material surface functionalization aims to add or expose reactive functional groups (e.g., amines, carboxyls, hydroxyls) that can form covalent bonds with the functional moieties of the biomolecules. Functionalization can be attained by plasma treatment,^218^ chemical etching,^214^ and oxidation.^212^ Biomolecules can be grafted either directly onto functionalized material surfaces^219^ or via linker molecules (spacers), such as silane^220^ or PEG molecules.^221^ The role of spacer molecules is to increase the distance between the biomolecule and the biomaterial surface, which preserves the proper conformation and spatial orientation of the biomolecule and prevents its denaturation, which can be caused by direct contact with a solid surface.^222^ Spacer molecules may also provide a wide range of properties that facilitate bone regeneration or implant integration. For example, heparin,^213,223^ a key protein involved in tissue repair, is often introduced onto a material surface to act as both a spacer and anticoagulation and anti-inflammatory factor.^224^ Similarly, PEG,^221^ known for its ability to reduce nonspecific protein binding, can be used as a spacer molecule, providing an antifouling effect *in vivo*. Chemical conjugation methods have been broadly employed for bone implant surface functionalization with biomolecules such as BMP-2,^213^ VEGF,^225^ the adhesion peptide RGD,^226^ and TGF-β.^214^ This immobilization strategy allows prolonged biomolecule presentation compared to physical adsorption.^227^ The main disadvantage of biomolecule chemical conjugation lies in the harsh conditions required for many coupling reactions (e.g., the use of toxic or denaturing reagents such as organic solvents) that may lead to reduced biomolecule activity.^228^ To minimize the loss in activity, an array of bioconjugation reactions that can be performed in aqueous solutions under mild conditions have been developed (e.g., conjugation via a hydrazone and oxime formation reactions^229^ or alkyne–azide coupling^230^).

The bonding of biomolecules to the biomaterial surface can also be achieved by biomimetic ligand–receptor pairing. The most popular conjugation method relies on the interaction between biotin and avidin, which is the strongest known noncovalent bond.^192^ This bond remains stable even under harsh conditions, including extreme temperatures and pH values, organic solvents, and other denaturing reagents. Due to the wide variety of commercially available biotinylated biomolecules, this approach has gained much attention in bone implant functionalization.^231^ It has been successfully applied to immobilize biomolecules such as BMP-2,^232,233^ fibroblast growth factor-2 (FGF-2),^233^ and fibronectin^234^ on the surface of biomaterials including gelatin/hydroxyapatite composites,^232^ hydroxyapatite-coated nanofibers,^233^ and titanium implants.^234^

The key benefit of biomolecule surface presentation is the direct exposure of immobilized factors to the host body fluids and cells infiltrating the biomaterial/tissue interface. This implant design may significantly accelerate the recruitment of immune cells and mesenchymal progenitor cells involved in the early stages of bone repair (inflammation and revascularization), which are considered the most critical for successful healing.

### Controlled/Sustained Release Delivery Platforms

Biomolecules used in bone regeneration often suffer from low stability and a short half-life *in vivo*.^235^ These issues are particularly prevalent in the case of protein biomolecules (e.g., BMPs, OPN, OC), the bioactivity of which depends on their 3D structure, and can be easily lost *in vivo* due to hydrolysis, proteolysis, and endocytosis.^236^ Disrupted protein structure not only leads to the loss of biological activity but also increases immunogenicity and the risk of implant rejection by the host.^235^ One approach to prolong the biological activity of biomolecules *in vivo* is to immobilize them in controlled/sustained release delivery systems. The most straightforward strategy to attain the sustained release of biomolecules is to physically encapsulate them within a matrix material (Figure 3C),^237^ e.g., a PEG hydrogel,^238^ gelatin,^238^ or collagen–hydroxyapatite matrix.^239^ In this approach, the biomolecule is added to the polymer solution prior to scaffold fabrication, which may be followed by the covalent cross-linking of biomolecules to the polymer matrix.^240^ The simplicity of this method has contributed to its widespread use in BTE to entrap biomolecules such as BMPs,^239^ PDGF, and VEGF.^238^ Biomolecules immobilized directly in the matrix are released by diffusion and polymer degradation. The biomolecule release rate depends fundamentally on the matrix porosity and degradation rate, as well as the affinity of the released molecules for the polymer molecules (e.g., chemical affinity, or affinity based on electrostatic interactions). Release kinetics can be controlled by properly selecting the matrix material (with the desired degradation rate and charge) and scaffold fabrication technique. However, this immobilization strategy offers relatively poor control over the biofactor delivery rate. Another drawback lies in the fact that biomolecules typically need to be added to the polymer solution prior to scaffold fabrication. Since scaffold manufacturing procedures often involve the use of toxic reagents (cross-linking agents, organic solvents) and nonphysiological conditions such as elevated temperature or UV irradiation, they can significantly diminish biomolecule activity.^227^

These limitations have driven research toward the development of protective micro- and nanocarriers that shield the immobilized biomolecules from unfavorable external conditions. Due to the simplicity of the fabrication, the most popular biomolecule delivery vehicles are spherical polymeric carriers such as microspheres,^241^ microcapsules, and nanospheres.^242−244^ Among spherical carriers, micro- and nanospheres made of biodegradable polymers such as poly(lactic acid) (PLA), PLGA,^239^ and alginate have found the broadest use in bone reconstruction.^245−247^ The kinetics of protein release from these carriers can be adjusted to a specific application by tailoring the particle size, porosity, and degradation rate, which can be attained by proper selection of the carrier material and fabrication method.^248^ An important disadvantage of biodegradable nano- and microspheres is the limited control over the biomolecule delivery rate and initial burst release.^249^ These issues can be resolved by coating the nano- or microspheres with a semipermeable membrane, which creates a significant barrier to biomolecule transport. Biomolecule release from core–shell microcapsules relies on molecular diffusion through membrane pores. The rate of diffusion is dictated by the size and distribution of the pores and membrane thickness.^250^ These properties can be modulated by altering the microcapsule manufacturing method,^251^ composition of the membrane-forming solution,^252^ and process parameters.^251^

Another type of biomolecule carrier used for controlled/sustained delivery of biomolecules facilitating bone regeneration is liposomes.^253,254^ Liposomes exhibit a high affinity for cell membranes, which ensures their easy uptake by cells.^255^ However, liposomes are highly susceptible to changes in pH and temperature, enzymatic degradation, oxidation, and hydrolysis,^256^ contributing to their relatively low stability in physiological environments. Another major drawback of liposomes is their tendency to aggregate, fuse, and leak the encapsulated molecules. Due to these limitations, the use of liposomes in bone regeneration is much less widespread than the use of polymer carriers.

Spherical biomolecule carriers can be introduced directly into bone defects^257−259^ or embedded in a polymer matrix, e.g., an injectable hydrogel^26,298^ or a solid scaffold,^260,261^ prior to implantation. Solid implants can be fabricated by suspending biomolecule carriers in a polymer solution that is subsequently molded^198^ or 3D-printed^262^ into the desired shape, followed by matrix solidification. Alternatively, the particles may be installed onto the implant surface; for example, via a solvent annealing technique based on seeding a carrier suspension in a volatile solvent onto the scaffold surface and solvent evaporation.^248^ Numerous studies have shown that incorporating biomolecule-loaded carriers into polymer matrices significantly prolongs the biomolecule release duration.^242^ As a result, bioactive agents can be released over extended periods ranging from weeks^257,258^ to months,^248^ greatly improving their capability to induce bone formation.^257−259^ Scaffolds containing spherical particles also exhibit considerably higher mechanical resistance than implants composed solely of a polymer matrix.^260−263^

Scaffolds incorporating polymer carriers loaded with biomolecules hold great promise for the development of biomimetic tissue constructs. To create a tissue construct, biomolecule carriers can be incorporated into a scaffold seeded with cells (e.g., progenitor cells). Biomolecules released from the carriers modulate cell behaviors, including their migration, adhesion, differentiation, and proliferation. For example, microspheres loaded with BMP-2 or IGF-1 incorporated into hydrogel scaffolds induce osteogenic differentiation of MSCs entrapped in a gel.^264^ Microspheres loaded with proangiogenic factors (e.g., VEGF and FGF) have been utilized to promote scaffold vascularization in the fabrication of prevascularized bone implants.^265,266^

Significant progress in biomimetic bone construct engineering has been made since the introduction of 3D printing. 3D printers can precisely control the spatial distribution of the biomolecule-loaded carriers within the scaffold and recreate the tissue-specific 3D organization of biochemical cues.^262^ In addition, multiple types of carriers (made of materials that degrade at different rates or carry different biomolecules, such as those that can act synergistically) can be combined into a single construct.^267^ This type of design can provide the sequential release of various biochemical signals in a spatially and temporally controlled manner that mimics the physiological release of biofactors during osteogenesis.

### Preprogrammed Release Delivery Platforms

Recreating the precise timing of biomolecule release during physiological bone healing remains one of the biggest challenges in bone regenerative medicine. To address this issue, preprogrammed release delivery platforms have been introduced. This type of platform is designed to deliver biomolecules at specific time points corresponding to their physiological release pattern. Among preprogrammed release delivery platforms, the pulsatile delivery systems^268−270^ have emerged as a promising approach to achieving precise temporal control over biomolecule release. These systems deliver therapeutic agents in pulses at predetermined intervals over an extended period (usually several months).^270,271^ Pulsatile release platforms may be based on multilayer or multicompartment constructs, where each compartment contains and releases the biomolecule at its own unique rate. Particular compartments can be made of different matrix materials (e.g., biodegradable polymers such as PLGA,^272^ gelatin,^271^ poly(4-vinylpyridine), alginate,^272,273^ PLLA^274^) with different degradation rates. The degradation of each compartment leads to a burst (pulse) of bioactive agents. The lag time between pulses can be precisely tuned by varying the molecular weights of the polymers, combining materials with different degradation rates in various proportions (e.g., glycolic and lactic acid in a PLGA copolymer),^271^ or adjusting the size/thickness of particular compartments. Another approach to pulsatile release is based on implantable microchip devices made of several reservoirs containing discrete doses of bioactive agent(s). The sequential release from each reservoir may be attained by sealing the reservoir with biodegradable PLGA membranes with various compositions^270^ or preprogramming the chip to open particular reservoirs at predetermined time points. Reservoir openings can also be triggered remotely using wireless communication.^275^

Preprogrammed delivery devices can be employed to provide accurate doses of single or multiple bioactive molecules. Single biomolecule dosing is particularly useful in the case of biofactors, the effect of which depends strongly on administration frequency. An example of such a biomolecule is PTH, which is used to stimulate bone formation in the treatment of osteoporosis. PTH can act anabolically (promoting bone formation) when administered intermittently^276^ and catabolically (leading to bone degradation) when administered continuously.^277^ For pulsatile PTH delivery, devices composed of multiple layers of biodegradable polymers have been proposed.^268,269,274^ For example, Dang et al. developed a pulsatile PTH delivery device consisting of alternating alginate layers loaded with the drug (PTH) and polyanhydride (PA) isolation layers that did not contain the drug.^269^ Such a device can release daily PTH pulses, upon the gradual degradation of its subsequent layers, for up to several weeks, after which the body completely resorbs the device without the need for implant removal. This system has been demonstrated to have a superior ability to induce new bone formation and much fewer side effects than conventional therapy that relies on daily systemic PTH injections. Another platform for PTH delivery, an implantable silicon microchip that releases drugs by wireless control, has successfully passed safety and efficacy evaluations in clinical studies.^275^ This type of device may provide a valuable alternative to the current FDA-approved PTH therapies in the near future.

The second goal of preprogrammed delivery systems is to allow the sequential release of multiple biomolecules involved in natural bone regeneration, including osteogenic, immunomodulatory, and proangiogenic factors such as BMPs,^272,278^ IGF-1,^279^ VEGF,^272,280,281^ PDGF, TGF-β1,^280^ interferon-γ, and interleukin-4 (IL-4).^282−284^ Sequential delivery of biomolecules has been shown to improve osteogenic outcomes in a number of *in vitro* and *in vivo* studies.^272,278,269,285^ Based on the accumulating evidence that the current clinical approaches based on high biomolecule concentrations cause numerous side effects,^286^ the preprogrammed devices that are currently under development have been designed to release bioactive agents at much lower doses (e.g., 6.5 μg of BMP-2 vs 6–12 mg in INFUSE Bone Graft).^227^ Low-dose devices, therefore, can potentially resolve the safety concerns of the regenerative bone therapies currently employed in clinical practice.

### Stimuli-Responsive Delivery Platforms

Another group of biomolecule delivery platforms is stimuli-responsive systems.^196,287^ These systems are able to deliver biomolecules on demand in response to specific stimuli, which can be categorized as physiological signals coming from the patient’s body (e.g., temperature, pH, body fluid composition, oxygen concentration, etc.^288,289^) and external stimuli such as exposure to ultrasound,^290,291^ near-infrared light,^292^ or electric^293^ or magnetic fields.^294^ The main aim of stimuli-responsive release is to achieve time- and site-specific drug delivery, which can effectively eliminate the systemic side effects of therapy.

Delivery platforms triggered by physiological stimuli do not require exposing the patient to external factors and are therefore considered safer and more convenient. For this reason, physiological stimuli-responsive platforms have been more extensively explored in bone regeneration than external stimuli-responsive platforms.^295^ An example of a system triggered by a physiological stimulus is polyelectrolyte microbeads (dextran methacrylate-AMPS microbeads) that release PTH in response to an increase in the Ca^2+^ concentration, which occurs in patients with osteoporosis due to bone loss.^296^ A different strategy for physiological stimuli-mediated release is based on the cleavage of the material encapsulating the biomolecule by enzymes naturally occurring in the bone ECM, such as metalloproteinases (MMPs) or collagenases.^297,298^ Since most synthetic biomaterials are not susceptible to enzymatic degradation, to create enzyme-sensitive delivery systems, the matrix material needs to be functionalized with enzyme cleavage sites. This can be achieved by chemically modifying the matrix with molecules containing specific amino acid sequences that can be recognized by enzymes, such as cleavable oligopeptides^299,300^ or cross-linkers (e.g., bis-cysteine peptides).^297^ In the presence of proteases secreted by host cells at the implantation site, the cross-linker is cleaved, which results in cell-mediated degradation of the polymer matrix and release of the entrapped biomolecules. Enzyme-sensitive systems used in BTE mainly employ PEG derivative hydrogels^301^ and hyaluronic acid hydrogels.^302^ These systems have been utilized for the local delivery of GFs (e.g., BMP-2,^288^ VEGF^298^) and chemokines e.g., stromal cell derived factor-1α (SDF-1α).^302^

The next group of stimuli-responsive systems is temperature-sensitive delivery platforms. These platforms typically employ thermoresponsive polymers^303−309^ that undergo gel–sol or sol–gel transitions at body temperature. Gel–sol transition leads to the release of biomolecules immobilized in hydrogel implants or microspheres^309^ upon implantation. Materials that transition from a sol to a gel at 37 °C are being used as *in situ* forming injectable hydrogels that remain liquid at room temperature but rapidly solidify into a gel upon injection, allowing long-term drug release. The most widely used thermogelling polymers are based on poly(*N*-isopropylacrylamide)^310^ and polyester block copolymers.^311−314^ Due to their injectability and gelation under physiological conditions, these polymers have been broadly applied to deliver biomolecules such as VEGF^310^ and BMP-2.^312^

Among the platforms that are sensitive to physiological stimuli, pH-sensitive systems have elicited much interest in bone regeneration. These platforms are based on materials that undergo a sol–gel transition, degradation, or volume change (swelling/shrinking) in response to changes in pH. For instance, at the desired pH, polymers may transition from a tightly packed to an expanded state,^315^ which leads to polymer swelling, liquefaction, and drug release. The pH range that triggers phase transition can be tailored to a specific target site by incorporating ionizable groups with specific p*K* values, that match the desired pH, into the polymer molecules.^315^ pH-sensitive materials employed in BTE include, among others, poly(NIPAAm-*co*-AAc) hydrogels,^316^ alginate/chitosan polyelectrolyte complexes,^317^ and chitosan^318,319^ and transition either at physiological pH (∼7.4) or under the acidic conditions (pH 5–6) found in healing tissues.^320^ Numerous pH-responsive platforms have been developed for the on-demand delivery of biomolecules such as BMP-2,^317^ VEGF, EGF,^292^ and dexamethasone.^318^ For greater control over biomolecule delivery, temperature- and pH-sensitive polymers can be combined into dual stimuli-responsive platforms that are sensitive to both pH and temperature.^320^ pH- and thermosensitive hydrogels can be fabricated by adding pH-responsive end groups to thermosensitive block copolymers. Such an approach has been employed to generate dual-responsive poly(ε-caprolactone-*co*-lactic acid) (PCLA)/PEG hydrogels for BMP-2 delivery.^321^ Combining two mechanisms to control drug release ensures highly specific dosing within a narrow range of physiological conditions.

### Gene Delivery Platforms

With advancements in genetic engineering, gene delivery platforms have been proposed as a new approach to release biomolecules accelerating bone regeneration.^322,323^ Gene delivery aims to upregulate the synthesis of biofactors involved in bone regeneration or silence signaling pathways that inhibit osteogenesis^324,325^ locally in the bone defect. The most common strategy for gene delivery is transfection of the target cells, which can be either host cells infiltrating an osseous defect or foreign cells transplanted into the lesion. Introducing genes encoding osteoinductive factors into cells allows their continuous expression and sustained release for extended periods, which may resolve the issue of the short half-life of biomolecules. Moreover, biofactors synthesized directly at the regeneration site in their native form display higher activity than exogenous recombinant proteins. Gene transfection is performed using vectors (viral or nonviral) that carry the foreign gene into the cell. The key advantage of viral vectors is their high transfection efficiency. Viral vectors (mostly adenoviral and retroviral vectors) have been broadly used for the local delivery of genes encoding osteoinductive agents such as BMPs,^326,327^ VEGF,^328,329^ LIM mineralization protein-1 (LMP-1),^330^ and cyclooxygenase-2.^331^ Viral vectors can be introduced into the bone defect directly by injection of a viral particle suspension^326^ or by implantation of a polymer matrix incorporating the vector.^332,333^ It has been shown that gene delivery using viral vectors leads to high gene expression levels in bone defects over a period of several weeks (typically 4–6 weeks),^334^ which accelerates bone healing considerably.^326,335,336^ However, viral vectors raise serious safety concerns regarding the activation of the host immune response as well as tumor formation due to the risk of random insertion of the transferred gene into the host genome.^337^

To overcome these issues, nonviral vectors have been proposed as safer alternatives to viral vectors. Nonviral vectors may take the form of plasmids,^338^ which are small circular pieces of free DNA carrying the transgene or other forms of nucleic acids such as cDNA,^91^ siRNA^324^ or microRNAs,^344^ (miRNA), which are small noncoding RNAs able to post-transcriptionally regulate pathophysiological signaling pathways via degradation of mRNA or inhibition of translation.^339^ Plasmids have been demonstrated to effectively deliver the PTH,^340^ VEGF,^341^ and BMP-4^342^ genes into host cells and successfully induce new bone formation. However, since naked DNA is easily degraded by nucleases, high doses are typically required to exert relevant therapeutic effects. To preserve the integrity of the transferred genes, plasmids,^343^ or other forms of nucleic acids such as cDNA,^91^ miRNA,^344^ and siRNA^324^ can be incorporated into protective polymer matrices called gene-activated matrices (GAMs, e.g., collagen sponges,^342,345^ collagen/calcium phosphate scaffolds,^340^ and triacrylate/amine-gelatin constructs^346^) or gene delivery vehicles such as liposomes^347,348^ or polycation-based nanoparticles. Liposomes used for gene delivery are usually based on cationic lipids, which spontaneously form complexes with nucleic acids due to the electrostatic interactions between the positively charged lipids and negatively charged nucleic acid molecules. The resulting lipoplexes protect DNA from degradation and are easily taken up by cells via endocytosis.^349^ Liposomal vectors have been demonstrated to effectively deliver biomolecule genes (e.g., VEGF and BMP-2) into bone defects.^91,350^*In vivo* studies have shown that the host cells surrounding an osseous lesion take up liposomes carrying BMP-2 cDNA and effectively express the transgene for up to 4 weeks, leading to enhanced bone formation. Nonetheless, the significant drawbacks of liposomes are the poor stability of lipoplexes in physiological fluids, their rapid clearance from the target site, and their tendency to aggregate.^351^ A variety of strategies have been developed to improve the performance of liposomes as gene carriers. Modifications of the liposome physicochemical properties, including size, charge, lipid composition, lipid-to-DNA ratio, and chain length, have been shown to increase liposome stability and cellular uptake.^253^ To allow tissue-specific gene delivery, liposomes have been modified with functional groups with a high affinity for bone (e.g., pyrophosphate^352^ or bisphosphonate groups^254^) or ligands that bind to specific receptors on the surface of the target cells (e.g., peptides, antibodies, or aptamers^351,353^). To prolong the retention of liposomes at the implantation site, liposomes have also been encapsulated in hydrogels,^354^ core-shell nanofibers,^355^ and microspheres.^344^

Another type of nonviral vector is polycation-based nanoparticles. Polycations such as polyethylenimine (PEI), poly-l-lysine (PLL), and chitosan^356,357^ have the ability to form complexes with nucleic acids due to their positive charge. During complex formation, the genetic material is condensed into nanostructures called polyplexes. The cationic regions of the polyplexes easily bind to negatively charged cell membranes, which promotes their uptake and contributes to the increased transfection efficiency compared to naked plasmids.^358^ However, the polycationic regions of polyplexes can disrupt the integrity of cell membranes, resulting in cytotoxicity toward host cells.^359^ To reduce this effect, the surface properties of polyplexes may be altered by chemical modifications, such as acetylation^360^ or carboxyalkylation.^361^

Each biomolecule delivery platform described above has its advantages and disadvantages. Current research has focused on combining the advantages of the systems developed to date into a single platform. The field of tissue engineering is currently progressing toward multicomponent systems comprising multiple types of biomolecule delivery vehicles.^362^ An interesting example of such a platform is a two-stage delivery system for the local delivery of miRNA (microRNA) that activates the osteoblastic activity of endogenous stem cells.^344^ This platform is composed of nanosized core–shell miRNA/polyplexes encapsulated in biodegradable polymer microspheres attached to an NF polymer scaffold. Such a design ensures high transfection efficiency due to the use of polyplexes. Encapsulating polyplexes within microspheres allows their release to occur in a controlled and sustained manner, while the attachment of microbeads to the NF scaffold enables their proper spatial distribution and effective fixation at the implantation site. This combination translated to greatly improved therapeutic effects in osteoporotic mice. The volume of the new bone formed upon implantation of this scaffold was six times higher than that in the group treated with naked miRNA.^344^ To allow further advancements in the field, the most recent studies aim to combine multicomponent scaffolds with different classes of signaling molecules and cells (particularly stem cells) that perform diverse biological functions during osteogenesis into a single biomimetic bone construct.^268^ To drive the progress of bioactive bone implants toward clinical translation, future research will need to resolve the issue of recreating the bone-specific spatial organization of biomolecules and cells within such constructs.

## Challenges and Solutions in the Development of Bioactive Materials for Bone Regeneration

Bioactive materials designed to deliver biomolecules to bone defects have contributed to significant progress in the field of bone regeneration and reconstruction. Multiple studies have confirmed that these materials can effectively accelerate new bone formation and, more importantly, engage in a complex biochemical dialogue with the host cells. Over the last several decades, the materials applied in bone regeneration have evolved from simple bioinert bone substitutes to highly advanced bioartificial systems able to both provide the mechanical support and respond to signaling factors secreted by the surrounding tissues. Currently researched bioactive implant materials have the potential to address key safety concerns of the current FDA-approved clinical approaches to bone healing based on BMP-2, i.e. the adverse side effects caused by supraphysiological BMP-2 concentrations.^211^

The limitations of current strategies for the treatment of bone defects have driven research toward the development of the biomolecule delivery platforms that would contain lower doses of biomolecules^196^ and provide a more precise control over their release. Despite promising results from research studies, so far only a few systems have reached the clinical setting. INFUSE Bone Graft from Medtronic, which is a collagen sponge soaked with recombinant BMP-2, is currently the only FDA-approved BMP-based product used in clinical practice. The vast majority of bioactive materials for bone regeneration applications remain at *in vitro* or animal testing stage, as they suffer still from relatively limited control over the biomolecule release rate. One of the approaches to resolving the issue of rapid biomolecule release (e.g., burst release) relies on multicomponent composite materials incorporating biomolecule-loaded carriers such as microspheres,^241^ core–shell microcapsules,^363^ or nanospheres.^242−244^ Biomolecule carriers provide much more precise control over the release kinetics of the therapeutic agent and can greatly prolong the duration of biomolecule release.^242^ As a result, multicomponent materials can deliver biomolecules gradually over extended periods ranging from weeks^257,258^ to even months,^240^ which considerably improves their capability to induce bone formation.^257−259^

Another key challenge in the field of bioactive bone implant materials is tailoring the timing and order of biomolecule release to release patterns occurring in the physiological bone healing. This challenge can be addressed by the stimuli-responsive delivery platforms,^196^ which release the biomolecules on-demand in response to specific stimuli (e.g., host cell-driven degradation of the polymer matrix) or preprogrammed delivery platforms secreting biomolecules at specified time intervals (e.g., implants releasing therapeutic agents by wireless control^274^).

An important factor hindering further advancements in bioactive materials for bone regeneration is low stability and the short half-life of biomolecules *in vivo*. The direction of research that seems the most promising in overcoming this problem is developing gene delivery platforms that allow *in situ* expression of factors promoting bone healing.^323,339^ These systems can effectively eliminate the problem of rapid loss of biomolecule activity at the target site by providing its continuous expression and sustained release locally in the bone defect.^326−330^

More studies are also necessary to improve control over the spatial distribution of the biomolecules or/and immobilized cells within the bioactive materials. The technology that can contribute to significant progress in this area is 3D bioprinting.^364^ This technology may enable manufacturing of personalized bone grafts combining multiple types of cells and materials loaded with different bioactive factors into a single platform. It is expected to allow us to recreate tissue-specific 3D organization of biochemical cues and cells within biomimetic bone tissue constructs in the near future. The development of cell-loaded bioactive materials able to sequentially deliver multiple biomolecules in a spatially and temporally controlled manner would represent a significant milestone in our progress toward smart biomaterials for bone regeneration applications.

As mentioned above, bone is a very complex multifunctional connective tissue whose properties allow it to perform several highly specialized functions in the human body. To serve its structural purposes and protect the vital organs (e.g., rib cage or braincase), bone has to be resilient. On the other hand, bones need to be stiff to provide the proper reaction to muscle contractions and withstand the applied forces (load). Moreover, bone remains a reservoir of minerals, particularly calcium and phosphate, and it provides niches for many cell types, including crucial progenitor and multipotent cells. To effectively carry out all of these tasks, the skeleton exists in a dynamic equilibrium characterized by continuous osteoclast-mediated bone resorption and osteoblast-mediated bone deposition. These highly orchestrated and simultaneous processes result in an imperceptible change in a bone mass called bone remodeling.^365^

Recently, the majority of studies on bone remodeling at the cellular level have focused on the roles of mature osteoblasts and osteoclasts and their respective precursor cells. It is worth noting that when mediating bone remodeling, there is growing recognition of the roles of two other types of cells found in bone, namely, osteocytes and bone lining cells. Osteocytes are mechanoreceptors derived from osteoblasts that remain trapped in the matrix.^366^ It has been proposed that osteocyte programmed cell death initiates the bone remodeling.^367^ The role of bone lining cells remains quite unclear and requires future investigation. However, it has been postulated that these cells play a role in the coupling of bone resorption to bone formation.^368^ It has also been confirmed that immune cells are capable of producing factors that both aid and suppress osteoclastogenesis. An altered balance between the expression of stimulating or suppressing factors will have an impact on bone homeostasis.^367^

Despite the unique capacities of self-regeneration and self-remodeling, several musculoskeletal diseases, such as osteogenesis imperfecta, osteoarthritis, osteomyelitis, and osteoporosis, can affect the physiological functions of bone tissue, which may have consequences on the quality of life of a patient. Furthermore, such diseases, combined with traumatic injuries, orthopedic surgeries, or primary tumor resection, may result in the damage and degeneration of tissues.^369^

One condition that comes with an increased risk of fracture in response to minimal or low velocity force and impaired bone regeneration is osteoporosis. Osteoporosis is defined by a decrease in bone strength due to lower bone density. In general, the areas most prone to fractures are the nonvertebral areas. These sites are characterized by bone that is composed of mainly compact or cortical tissue that accounts for 80% of the total bone mass in an adult skeleton, while trabecular tissue makes up the remaining 20%.^370^ Peak bone mass is reached at the end of the third decade of human life. After this point, the balance between bone formation and bone resorption is impaired, with a relative increase in bone resorption that leads to net bone loss. According to recent research, after the age of 65, the majority of bone loss is cortical bone loss. Nonetheless, the postmenopause bone loss observed in women is mainly trabecular bone loss. The consequence of the imbalance between bone formation and resorption and the subsequent deterioration of the skeletal microarchitecture will result in the loss of bone tissue and bone strength.^371^

The basic diagnostic techniques that determine bone strength and lead to targeted intervention strategies in osteoporosis treatment include BMD measurements, bone geometry determinations, evaluations of bone microstructure, extent of bone mineralization, and examinations of the properties of the bone matrix or the presence of a fragility fracture.^372^ Osteoporotic fractures are associated with serious consequences, such as a diminished quality of life, decreased functional independence, and increased morbidity and mortality. Therefore, there is a great need to improve diagnostic strategies and optimize the prevention and treatment of osteoporosis.^367^

Taking these factors into consideration, an improved understanding of the pathophysiology of osteoporosis will result in better therapeutic and diagnostic procedures for this disease. It is worth noting, in light of the growing prevalence of osteoporosis and its association with the danger of trauma, discovering factors that can modulate the risk of osteoporotic trauma would significantly increase the number of people that qualify for treatment.^367^

Recently, the role of the immune system in the pathogenesis of osteoporosis has increasingly been recognized, prompting the emergence of the field of osteoimmunology. The immune system has been postulated to play an essential role in the etiology of bone disease by unbalancing the actions of bone-resorbing osteoclasts and bone-forming osteoblasts.^372,373^

Clinical examinations of autoimmune disease samples have demonstrated that autoantibodies can induce the differentiation and activation of osteoclasts and alter bone mineral content. The immunological causes of bone destruction appear to stem from inflammation and autoimmunity. For instance, independent risk factors for the development of bone erosions and osteoporosis in rheumatoid arthritis (RA) are autoantibodies such as rheumatoid factor (RF) and anticitrullinated protein (ACPA).^340,374,375^

RA is a chronic autoimmune inflammatory disease that is characterized by local bone erosion, joint space narrowing, and extra-articular manifestations caused by the production of two main autoantibodies, RF and ACPA, against common autoantigens that are widely expressed outside the joints. Severe cases of RA may result in periarticular osteopenia, systemic osteoporosis, and systemic bone erosion. Elevated inflammatory cytokines (such as TNF-α, IL-1, IL-6, IL-7, and IL-17) in RA are involved in bone destruction through the recruitment of osteoclast precursors to the bone environment, where they differentiate into mature cells. These inflammatory cytokines induce the overexpression of RANKL and decrease the levels of OPG (an alternate receptor of RANK), and this perturbation leads to increased osteoclastogenesis. Nevertheless, significant amounts of anti-inflammatory cytokines have also been reported to be present in RA joints. Cytokines, such as IL-10, IL-13, and TGF-β, negatively affect joint destruction and the inflammation associated with RA. In summary, chronic inflammation of the synovium and thus bone destruction in RA is caused by a complex network of inflammatory cytokines. Thus, therapies aimed at inflammatory cytokines and/or lymphocyte activation may modify RA treatment by blocking local and systemic inflammatory cascades and supporting the beneficial effects against bone and joint destruction.^376−378^

RA along with other inflammatory autoimmune diseases (systemic lupus erythematosus (SLE), ankylosing spondylitis (AS), and inflammatory bowel disease (IBD)) continue to be increasing public health problems worldwide. A better understanding of the mechanisms by which the inflammatory cytokine network induces chronic inflammation in autoimmunity will provide new therapeutic approaches to reduce bone destruction in inflammatory autoimmune diseases.^376^ Even though primary bone cancers are rare, bone often becomes a plausible niche for the metastatic spread of various cancers. Surgical, irradiation, or chemotherapy-based cancer removal does not generally guarantee complete clearance of all cancer cells. On the other hand, several cancer treatment options may induce bone loss, causing or enhancing osteoporosis in these patients. Remnant tumor tissue promotes the release of inflammatory cytokines and osteoclast activation, which in turn, drive the excessive degradation of transplanted bone tissue or bone-mimicking implants. Traditional resection and reconstruction cannot provide adequate bone healing and regeneration in such cases.^379^ Recently, magnetic field-responsive nanoparticles containing Fe_3_O_4_ were developed to kill cancer cells in response to external magnetic field sources by elevating the temperature of the tissues in contact with nanoparticles. The magnetic field application is safe for the end user and leaves the normal surrounding tissue untouched. Intriguingly, the application of Fe_3_O_4_ nanoparticles and application of magnetic hyperthermia enhanced bone regeneration by an unclear mechanism.^380,381^ Other types of intelligent, tumor-killing materials were developed based on the controlled release of cytotoxic butyrate or Fe-CaSiO_3_, which can be further enhanced by photothermal therapy.^382,383^ Such therapies are characterized by noninvasiveness and high controllability, showing great promise in bone tissue regeneration applications.

Before planning a therapeutic strategy aimed at treating specific diseases, it is important to recognize that bone regeneration is highly dependent on the formation of a new blood vessel network. The efficiency of the formation of new bone broadly depends on the growth rate and the extent of the blood vessels. Thus, when reconstructing large bone defects using cell-based tissue engineering, it is important to improve the strategies employed for bone vascularization. This is of particular importance when seeding cells in the central region of the scaffolds, as cells may die due to insufficient access to nutrients and oxygen. Traditional methods employed for engineering vascularized bone directly target the defect site, thus optimizing the healing process. Among them, we can include culturing BMSCs, endothelial progenitor cells, endothelial growth factors, and FGFs, along with endothelial cell monoculture and the coculture of endothelial cells and bone-forming cells. Despite its potential, the clinical applications of tissue-engineered vascularized bone are still very limited. To determine the appropriate release kinetics of GFs and establish new tissue engineering scaffolds for inducing angiogenesis and bone morphogenesis, further research is needed. Finally, the newly designed scaffolds should also support the differentiation of stem cells into vascular precursors for osteogenesis.^384^

## Conclusions

Globally, an estimated 175 million people suffer from bone fractures yearly, and many require implantation surgeries to fill in bone defects. Stimulation of the regeneration process of extensive bone tissue defects is challenging, and autologous graft is often excluded as an option to treat affected individuals. Significant defects can be the cause of the development of disabilities.

The growing field of bone replacement material engineering aids the healthcare systems in treating complex and extensive cases of bone loss. Currently, many innovative biomedical approaches are being tested worldwide to develop advanced bone regeneration strategies. The most advanced scaffolds are the fruits of the work of multidisciplinary research groups involving material chemists, material engineers, biologists, and medical professionals. Since the legislation process is demanding regarding product biosafety and biocompatibility, most advanced bone-replacement scaffolds are at various stages of design, preclinical, or clinical studies. A separate group of recipients are people whose bone loss is associated with degenerative diseases and cancer. Even more sophisticated and personally dedicated advanced solutions are needed in such cases, remaining the major challenge in the field of bone regeneration.

The “perfect scaffold” for treating bone defects would be made of biomaterials that mimic the properties of the natural bone, ideally containing living and dividing progenitor cells in its structure. Such an environment would support not only the growth and differentiation of bone tissue but also its vascularization and even innervation, which requires the presence of numerous signaling molecules, growth factors, and metabolites found in natural bone. The complexity of such a system causes problems in the fabrication of the perfect scaffold and in ensuring its stability and viability. The methodological advances presented in our review show that the scientific world is getting closer to formulating a recipe for producing a near-perfect implant. Functionalization of modern implants with osteoconductive fractions of hydroxyapatites, collagens, growth factors, bioactive peptides, and metabolites is entirely feasible thanks to overcoming technological gaps in material fabrication approaches.

Looking at the popularity of this research topic and the extensiveness and complexity of scientific approaches, we are convinced that, in the next few years, perfect implants will enter the healthcare market. These solutions will present ideal mechanical properties, be bioresorbable, and be fully replaced with the patients healthy and adequately vascularized bone tissue.
